# Phosphonium Poly(Ionic Liquid) Electrolytes for Fast Lithium-Ion Conduction

**DOI:** 10.1021/jacs.6c02428

**Published:** 2026-04-29

**Authors:** Alejandro Herranz Berzosa, Kewei Cai, Gabriele Lingua, Daniele Mantione, lñaki Santos, Federica Santino, Maria Forsyth, Fangfang Chen, David Mecerreyes

**Affiliations:** † POLYMAT, 2104 University of the Basque Country UPV/EHU, Avenida Tolosa 72, Donostia-San Sebastian 20018, Spain; ‡ Institute for Frontier Materials, Deakin University, Burwood, Victoria 3125, Australia; § IKERBASQUE, Basque Foundation for Science, Bilbao 48011, Spain

## Abstract

Polymer electrolytes based on ionic liquids provide a safe solution for future solid-state high-energy-density batteries. In this work, we report a novel class of solid polymer electrolytes for fast lithium-ion conduction based on phosphonium poly­(ionic liquid)­s (poly­(IL)­s). First, a new family of poly­(diallyldimethylphosphonium) poly­(IL)­s was synthesized by cyclopolymerization of diallyldimethylphosphonium iodide following anion exchange with different sulfonamide-based anions. The characterization of the poly­(IL)­s confirmed the formation of a stable 5-member ring phosphonium cationic polymer backbone. Then, solid polymer electrolytes (SPEs) were prepared by doping the phosphonium sulfonamide poly­(IL)­s with different contents of lithium fluorosulfonamide salt (LiFSI). The nature of the ionic interactions and lithium transport of SPEs with different anion systems was deeply investigated by FTIR, DSC, ionic conductivity, solid-state ^7^Li NMR, and molecular modeling simulations. Polymer-in-salt phosphonium SPEs showed excellent properties, with high ionic conductivities up to 1.5 × 10^–3^ S cm^–1^ at 80 °C, high lithium transference numbers up to 0.7, and a wide electrochemical stability window superior to state-of-the-art SPEs. Finally, we showed that phosphonium poly­(IL) electrolytes allowed successful Li^+^ plating/stripping as a solid electrolyte in lithium metal symmetrical cells, showing constant overvoltage limited to 0.06 V working at 0.1 mA cm^–2^ current density at 60 °C, and stable cycling without signs of short-circuiting during 200 cycles at 40 °C.

## Graphical abstract

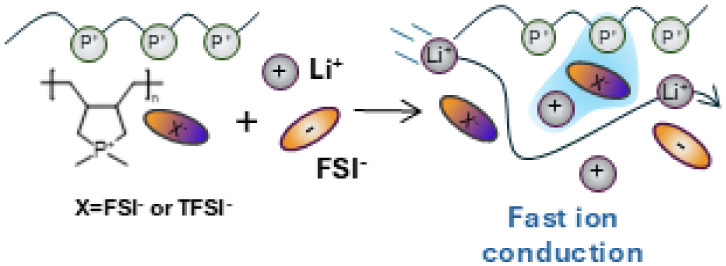

## Introduction

Solid polymer electrolytes (SPEs) provide a safe solution for future solid-state high-energy-density batteries. They have shown great potential as ion-conducting interlayers, enabling stable and efficient electrochemical reactions at metal anodes such as lithium and sodium. Historically,^1^ poly­(ethylene oxide) (PEO) has been the gold standard polymer matrix solid polymer electrolyte (SPE), engineered in different forms such as block copolymers, polymer brushes, or nanocomposites. In the last few years, different ionic polymers (anionic, zwitterionic, and cationic), called poly­(ionic liquid)­s (poly­(IL)­s), have emerged as the best candidates to prepare polymer electrolytes. These poly­(IL)-based SPEs are showing an excellent combination of high ionic conductivity and high Li^+^/Na^+^ transport number, along with superior electrochemical stability, which are critical factors to avoid degradation and polarization of the electrodes and premature battery failure in high-energy cells. For instance, sulfonamide-based anionic poly­(IL)­s, known as lithium single-ion conducting polymer electrolytes^2–7^ are a popular alternative due to their lithium transference number being close to one. On the other hand, polyzwitterions have recently appeared as an alternative that can self-assemble into superionic conductive domains, permitting decoupling of ion motion and polymer segmental rearrangement, showing high ionic conductivity and selectivity.^8,9^ More recently, cationic poly­(IL)­s have been shown to be excellent matrices for supporting high salt concentrations. These polymer-in-salt systems present fast alkali metal ion transport through structural diffusion, facilitating a high metal ion transference number.^10,11^

Among the different cationic poly­(IL)­s, the ones based on the ammonium polymer backbone poly­(diallyldimethylammonium) (polyDADMA) are very popular, both as polymer electrolytes to host different alkali salts and ionic liquids,^12^ as well as ionic binders for battery electrodes.^13,14^ The synthesis of the poly­(IL)­s is straightforward, starting from the commercially available poly­(DADMA) chloride polyelectrolyte or from the free radical cyclopolymerization of diallyl ammonium monomers. Thus, thermally and electrochemically stable poly­(DADMA) poly­(IL)­s with many different counter-anions, such as sulfonamides, dicyanamides, or phosphates, have been investigated.^15,16^ However, these attempts to improve and optimize its performance as a polymer electrolyte are reaching a limitation in ionic conductivity values and mechanical properties. While, for improving the mechanical properties, block copolymer design has been demonstrated as a successful strategy, the ionic conductivity still needs to be raised to support battery operation at or near room temperature^17,18^ For this reason, we looked back to ionic liquids (ILs) chemistry, where phosphonium ILs generally outperform their ammonium counterparts. Phosphonium cations are larger and more flexible than ammonium ones, allowing better ion mobility and weaker cation–anion interactions^20^ showing higher thermal stability, lower viscosity, higher ionic conductivity, and wider electrochemical windows. In a pioneering work, Long et al. anticipated that the phosphonium poly­(IL)­s showed lower glass transition temperatures (*T*_g_), higher thermal stabilities, and higher ionic conductivities than the ammonium analogs, and demonstrated their superior performance in demanding applications such as gene delivery.^20^ Interestingly, Butler and Berlin reported several decades ago the free-radical cyclopolymerization of phosphorus-containing diallyl monomers,^19^ but this route was not further explored for the synthesis of poly­(ILs).

In this article, we show the synthesis of phosphonium poly­(IL)­s for the preparation of SPEs with fast lithium-ion conduction for stable battery operation in a lithium metal cell. For this purpose, a new diallyl phosphonium halide ionic liquid monomer was designed, and its cyclopolymerization was investigated. Two different phosphonium poly­(IL)­s with sulfonamide counter-anions, TFSI and FSI, were synthesized and characterized. Solid polymer electrolytes (SPEs) were prepared by combining phosphonium poly­(IL)­s with LiFSI lithium salt, obtaining fully FSI anion or mixed FSI/TFSI anion electrolytes. The SPEs were fully characterized chemically and electrochemically through impedance spectroscopy to measure ionic conductivity, as well as FTIR, DSC, and solid-state NMR to investigate phase behavior and ion interactions. Furthermore, molecular dynamics simulations were carried out to provide more detailed insights into the structural environment and ion transport behavior. Finally, the ability of these phosphonium SPEs to enable lithium metal electrochemistry was investigated in a Li/Li symmetrical cell at low and moderate temperatures.

## Results and Discussion

### Synthesis and Characterization of Poly­(diallyldimethylphosphonium) Poly­(IL)­s

Scheme 1 presents the synthetic route to the diallyldimethylphosphonium monomer (DADMP) and its polymerization. For the synthesis of the diallyldimethylphosphonium iodide monomer ((DADMP)­I), methylphosphorous dichloride precursor is first synthesized following a previously reported procedure.^21^ The synthesis of the DADMP monomer is then carried out via a two-step reaction. In the first step, methylphosphorus dichloride reacts with 2 equiv of allylmagnesium bromide, and then, in a second step, a quaternization reaction is carried out by adding iodomethane to obtain the desired monomer. The synthesis is shown in Scheme 1a and detailed in the SI. The monomer was characterized by ^1^H, ^13^C, and ^31^P NMR to confirm the reaction and its purity (Figure S1). Next, free radical cyclopolymerization was carried out in aqueous media to form the poly­(diallyldimethylphosphonium) iodide (poly­(DADMP)­I) polyelectrolyte. The final sulfonamide phosphonium poly­(IL)­s, namely, poly­(DADMP)­FSI and poly­(DADMP)­TFSI, were obtained following an anion exchange reaction with LiFSI or LiTFSI, as reported in Scheme 1b.

**Scheme 1:**
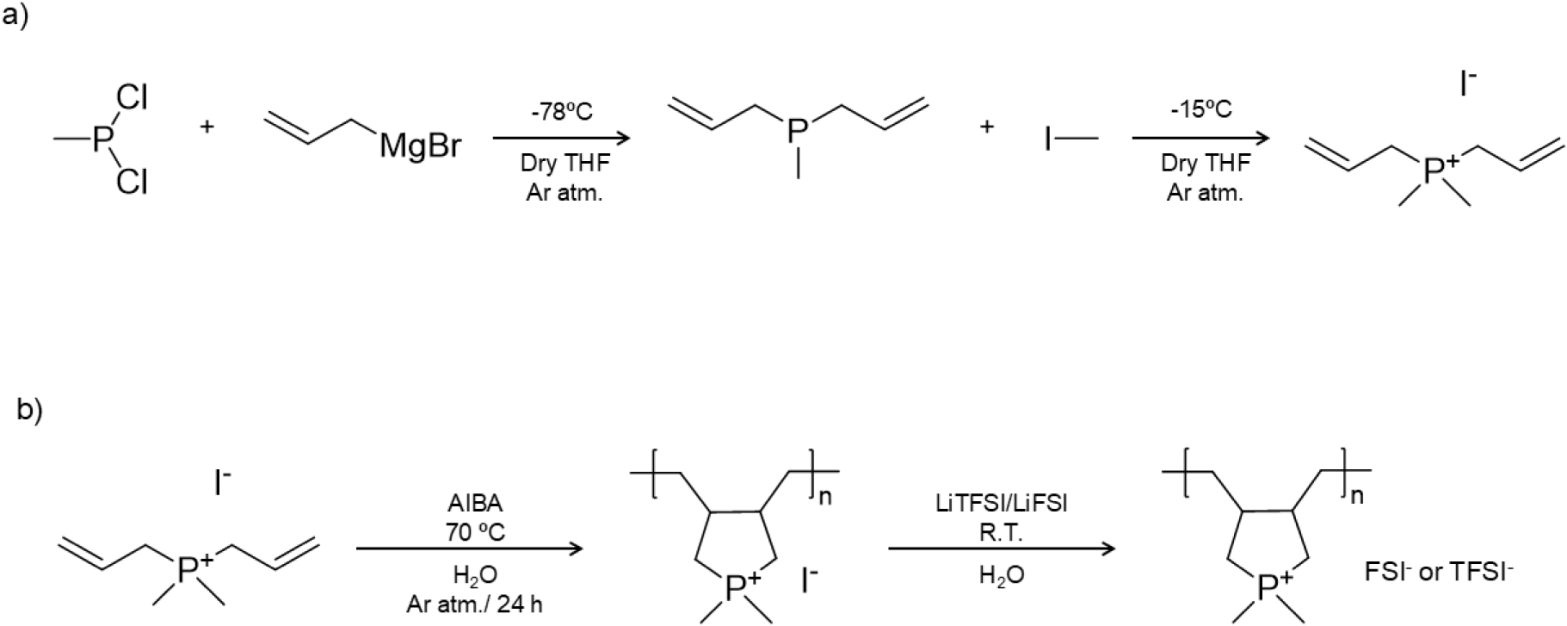
(a) Synthetic Route to Prepare the diallyldimethylphosphonium Iodide Monomer (DADMP)­I, and (b) The poly­(diallyldimethylphosphonium) Sulfonamide Poly­(IL)­s, Namely Poly­(DADMP)­FSI or Poly­(DADMP)­TFSI

After anion exchange, both poly­(DADMP)­FSI and poly­(DADMP)­TFSI were obtained as white powders. Due to their low solubility in common SEC solvents, the molecular weights (*M*_w_) of poly­(DADMP)­FSI and poly­(DADMP)­TFSI could not be determined. In contrast, the molecular weight of precursor poly­(DADMP)I was successfully measured by aqueous GPC. Figure S2 presents the GPC trace, revealing an *M*_n_ of 40 000 g/mol and a dispersity of 1.6, consistent with typical values obtained via free-radical polymerization.

FTIR measurements were also performed on poly­(DADMP)I to assess the presence of phosphoryl (P=O) groups, which could arise from the partial oxidation of the monomer during polymerization. The results, shown in Figure S3, reveal no signal corresponding to the P=O stretch at 1172 cm^–1^, as reported in the literature. The absence of this characteristic band confirms the stability of the monomer under polymerization conditions and indicates the formation of an unoxidized polymer.

Following the synthesis of both polymers, solubility tests were conducted to assess their solvent compatibility. The results, summarized in Supplementary Table 1 (Supporting Information), indicate that both polymers are soluble in ACN, DMSO, and NMP; partially soluble in THF, DCM, DMF, propylene carbonate (PC), and MeOH; and insoluble in H_2_O and hexane.

The chemical structure of the phosphonium poly­(IL) backbone was investigated first by NMR. The ^1^H NMR spectrum of poly­(DADMP)­FSI exhibits broad resonances characteristic of polymeric materials obtained by cyclopolymerization (Figure 1a). Signals at 0.9 and 1.1 ppm are assigned to the backbone methine and methylene protons, respectively.^22^ A resonance at 1.9 ppm corresponds to the methyl protons of the heterocycle, and a broad signal at 2.4 ppm is attributed to the methylene α-protons adjacent to the heteroatom. Poly­(DADMP)­TFSI displays nearly identical ^1^H NMR spectra (Figure S4), as is expected, since both materials have the same polymer backbone. Further structural information was obtained from ^13^C and ^31^P NMR spectroscopy (Figure S5). The ^13^C NMR spectra of poly­(DADMP)­FSI and poly­(DADMP)­TFSI show comparable features, including resonances at: 5–10 ppm (heterocyclic methyl carbons), 25 ppm (backbone methylene carbons), 30 ppm (backbone methine carbon), and 45 ppm (heterocyclic methylene carbons). Poly­(DADMP)­TFSI ^13^C NMR spectra also confirm the successful anion incorporation due to the presence of a multiplet at 120 ppm in the latter, assigned to the CF_3_ group of the TFSI anion. The ^31^P NMR spectrum shows, for both polymers studied, a signal at 22 ppm attributed to the phosphonium heteroatom. The appearance of a single signal indicates high purity of the compound. However, partial oxidation has been observed when the polymer is exposed to oxygen for extended periods of time. This behavior is further discussed in Supporting Information (Figure S7).

**Figure 1:**
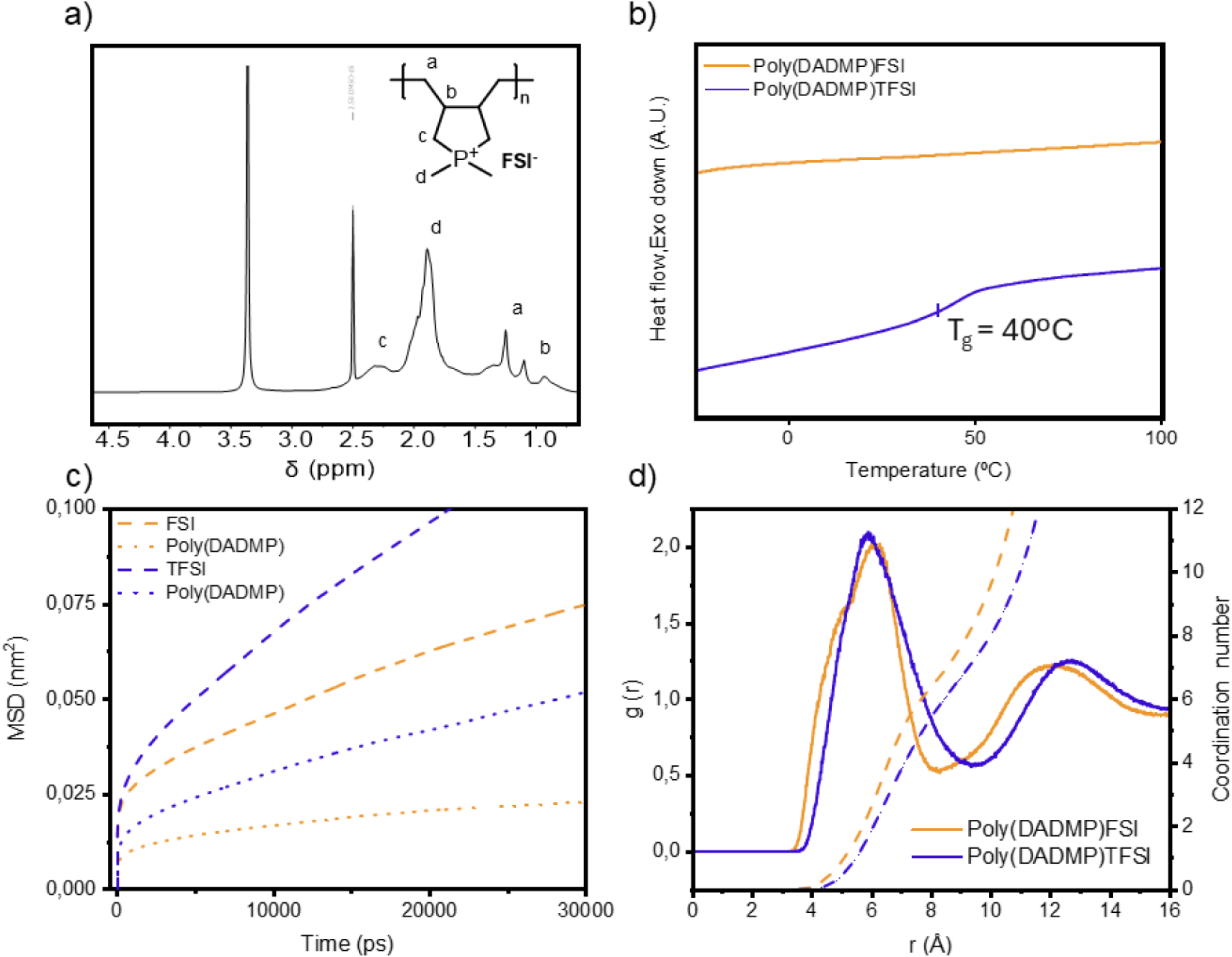
(a) ^1^H NMR of the poly­(DADMP)­FSI; (b) DSC of poly­(DADMP)­FSI and poly­(DADMP)­TFSI; (c) Mean square displacement of N atoms in anions and P atoms in poly­(DADMP)­FSI and poly­(DADMP)­TFSI; (d) RDF g­(r) of poly­(DADMP)­FSI and poly­(DADMP)­TFSI through the P atom in the polycation and the N atom anions.

Indeed, a comparative analysis of the ^1^H and ^13^C NMR spectra of poly­(DADMP)­TFSI and poly­(DADMA)­TFSI (Figure S6) reveals systematic upfield shifts for carbons and protons at the α-position with respect to the heteroatom for the phosphonium poly­(IL)­s. This behavior arises from the lower electronegativity and larger ionic radius of the phosphonium cation, with a resultant lower deshielding effect when compared with the ammonium counterpart.^23–25^ Indeed, the NMR comparison suggests that polymerization proceeds via cyclopolymerization with a ring-closing mechanism, yielding the intended phosphonium five-membered ring polymeric backbone structure similar to the well-known ammonium poly­(DADMA). The five-membered structure has also been confirmed with HSQC experiments presented in Supporting Information (Figure S8).

The chemical structure of the polymer, the presence of the counter-anions, and the successful ion exchange in the phosphonium poly­(IL)­s were verified by FTIR spectroscopy (Figure S9). Poly­(DADMP)­FSI displays bands at 1009 cm^–1^ and 1162 cm^–1^ corresponding to SO_2_ symmetric stretching vibrations, while poly­(DADMP)­TFSI shows bands at 1126 cm^–1^ and 1178 cm^–1^ assigned to SO_2_ symmetric stretching, along with an additional band at 1049 cm^–1^ attributed to the CF_3_ of the TFSI anion.^26^ These results confirm the presence of the respective counter-anions and the successful synthesis of phosphonium poly­(IL)­s bearing FSI or TFSI sulfonamide counter-anions.

Next, the thermal behavior of the poly­(IL)­s was investigated by DSC and TGA (Figure 1b and Figure S10, respectively). No clear glass transition (*T*_g_) was detected for poly­(DADMP)­FSI within the temperature range studied. This behavior is consistent with prior observations for ammonium poly­(DADMA)-based analogues, where the rigidity of the backbone does not allow *T*_g_ detection by DSC.^27^ Notably, the poly­(IL) material is a brittle solid, which suggests *T*_g_ above room temperature. In contrast, poly­(DADMP)­TFSI exhibits a clear *T*_g_ onset at 40 °C. This value is substantially lower than that of ammonium poly­(DADMA)­TFSI (116 °C reported in the literature from DMTA).^28^ The reduced *T*_g_ confirms the flexibility of the larger phosphonium cation and the weaker interionic interactions between the phosphonium polycationic backbone versus the ammonium one and the sulfonamide anions. On the other hand, poly­(DADMP)­FSI shows satisfactory thermal stability at 240 °C, while poly­(DADMP)­TFSI exhibits a higher decomposition onset near 400 °C (Figure S10). The reduced stability of the FSI-containing polymer is attributed to the intrinsic lability of the S–F bond. Interestingly, the decomposition of poly­(DADMP)­FSI occurs in two distinct steps, consistent with previous observations for poly­(DADMA)­FSI.^29^ Compared with their ammonium counterparts, the phosphonium-based neat polymers exhibit distinct thermal decomposition behaviors. Specifically, poly­(DADMP)­FSI shows a slightly lower decomposition temperature (240 °C) than poly­(DADMA)­FSI (300 °C).^30^ In contrast, poly­(DAMP)­TFSI and poly­(DADMA)­TFSI show similar thermal stability, decomposing at 400 °C. Overall, both materials exhibit excellent thermal behavior suitable for battery energy-storage applications.

Ionic conductivity measurements of the neat poly­(IL)­s have also been made using impedance spectroscopy to obtain a comparison of the anion dynamics in these new polymer scaffolds (Figure S11). At 100 °C, poly­(DADMP)­FSI exhibits an ionic conductivity of 6.5 × 10^–7^ S cm^–1^, while poly­(DADMP)­TFSI shows a higher conductivity of 2.6 × 10^–6^ S cm^–1^. At 40 °C, the ionic conductivity decreases to 8.1 × 10^–9^ S cm^–1^ for poly­(DADMP)­FSI and 5.4 × 10^–9^ S cm^–1^ for poly­(DADMP)­TFSI. It is interesting that, at higher temperatures, the ionic conductivity of the TFSI polymer is higher than the FSI analogue, whereas at low temperatures the FSI is more conductive. As expected, a change of the counterion affects the activation energy for ion transport, which is reflected in the change of slope in the ionic conductivity versus temperature plot. It is worth noting that, from a practical perspective, these ionic conductivity values of the poly­(IL)­s by themselves are insufficient for electrochemical applications, but they establish a baseline for further optimization with the incorporation of an alkali metal salt.

For a deeper study of the dynamics of the neat polymers, MD simulations (Figure 1c) were carried out on the phosphonium poly­(IL)­s. Supplementary Tables 2 and 3 show the details and parameters of the MD simulations, and the molecular dynamics procedure is also detailed in SI. The modeling results also show higher anion diffusion in poly­(DADMP)­TFSI at 80 °C. This can be attributed to two factors identified from our computational analysis. First, the volume expansion rate, i.e., Δ*V*/Δ*T* = [*V*(*T*) – *V*(*T*_g_)]/(*T* – *T*_g_) (Figure S12), is larger in poly­(DADMP)­TFSI compared to poly­(DADMP)­FSI above their simulated *T*_g_ (Figure S13), which contributes to increased free volume growth. Second, the radial distribution function (RDF) between the polycation phosphorus charge center and the N atom of the anion (Figure 1d) indicates a relatively bulkier solvation shell in the TFSI system, which may facilitate easier TFSI dissociation.

### Ionic Structure and Dynamics in Phosphonium Poly­(IL)-In-Salt Solid Polymer Electrolytes

Poly­(DADMP)­FSI:LiFSI and poly­(DADMP)­TFSI:LiFSI SPEs were prepared by adding controlled amounts of LiFSI, ranging from 1:1 up to 1:3 poly­(IL):LiFSI, in a solution of anhydrous acetonitrile, with subsequent solvent evaporation and extensive drying of the SPEs, as illustrated in Scheme 2. The SPE preparation and nomenclature specifications are described in the SI. The solid electrolytes exhibited a viscous, brownish appearance and a sticky texture, which made their handling and coin cell assembly particularly challenging. Figure 2a and b show the ionic conductivity values obtained for the poly­(DADMP)­FSI:LiFSI and poly­(DADMP)­TFSI:LiFSI SPEs, respectively. Poly­(DADMP)­FSI:LiFSI electrolytes show a remarkably high ionic conductivity across all poly­(IL):salt ratios, with the poly­(DADMP)­FSI:LiFSI 1:1.5 composition having the highest ionic conductivity value of 1.5 × 10^–3^ S cm^–1^ at 80 °C, 9.9 × 10^–4^ S cm^–1^ at 70 °C, and 1.5 × 10^–4^ S cm^–1^ at 30 °C (Figure 2a). On the other hand, for the mixed system, poly­(DADMP)­TFSI:LiFSI 1:2.5 shows the highest ionic conductivity of 5.3 × 10^–4^ S cm^–1^ at 80 °C, 3.4 × 10^–4^ S cm^–1^ at 70 °C, and 2.1 × 10^–5^ S cm^–1^ at 30 °C (Figure 2b). Surprisingly, the poly­(DADMP)­TFSI:LiFSI electrolyte shows lower ionic conductivity than poly­(DADMP)­FSI:LiFSI, in contrast to some previous reports, which suggested that mixed anions lead to higher alkali metal ion transport.^11,31–34^ However, most of the studies related to mixed anion systems were carried out in liquid and/or gel-polymer electrolytes, where conductivity modulation by the polymer backbone is not a factor.^31–33,35,36^ In these highly concentrated poly­(IL)-in-salt systems, it has been shown that cocoordination of the counteranion between the polymer backbone and the alkali metal plays a crucial role in determining high metal ion transport^34^ and therefore, in the presence of mixed anions, where there will be competition for coordination by the two different anions, lithium transport may be adversely affected depending on the nature of the ions. Certainly, there has been only limited investigation into understanding how polycation–anion interplay could influence ion structure and transport in these systems.

**Scheme 2:**
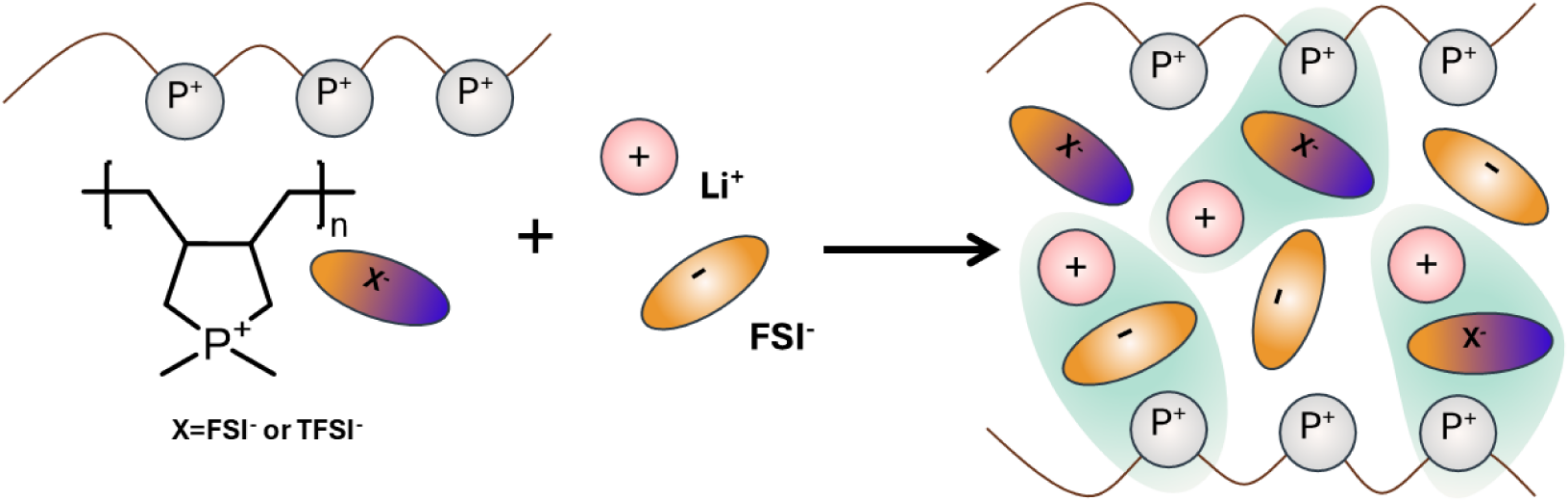
Sketch Representing the Composition of Solid Polymer Electrolytes (SPEs) and Its Ionic Conductivity

**Figure 2:**
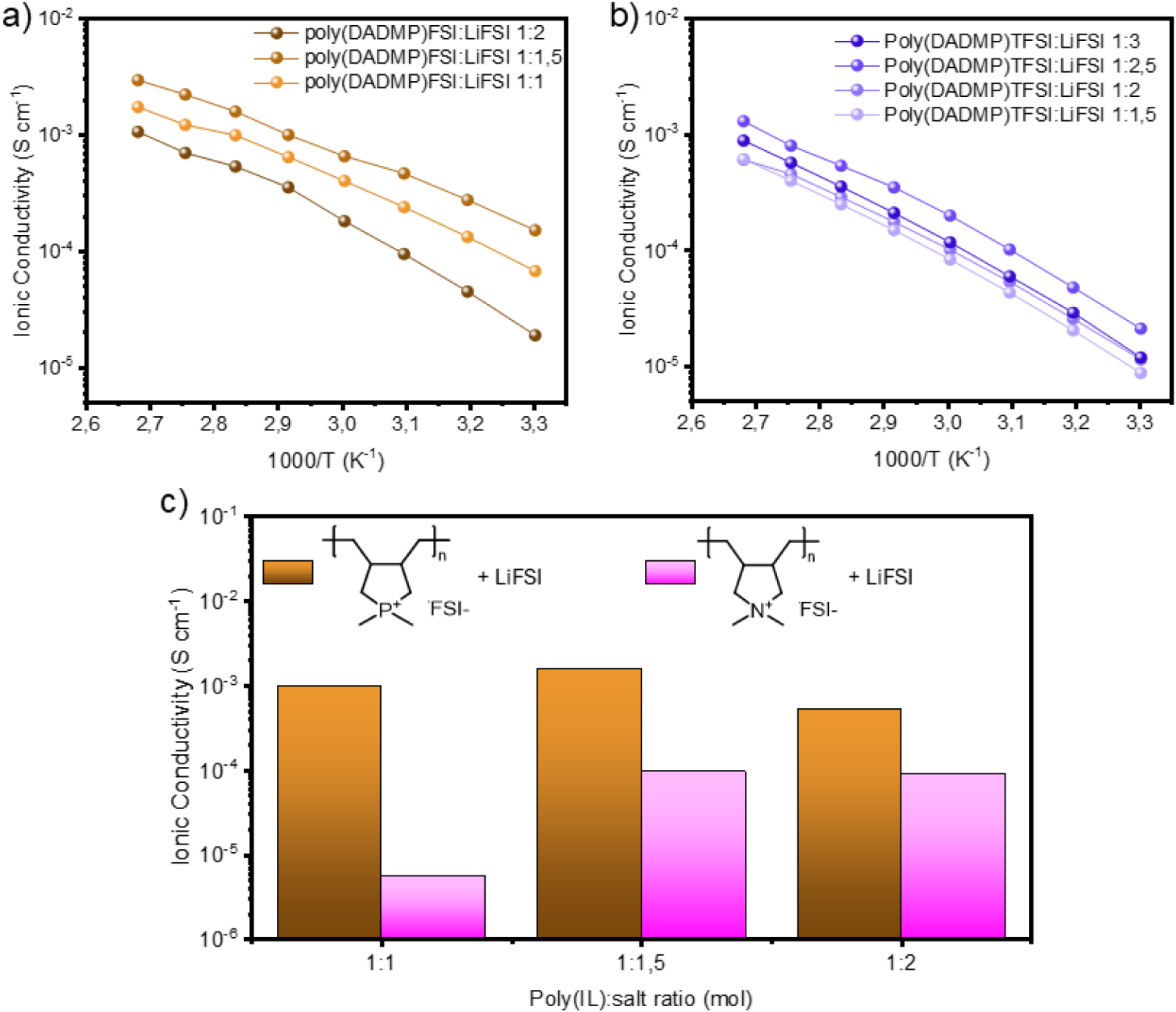
(a) Poly­(DADMP)­FSI:LiFSI and (b) poly­(DADMP)­TFSI:LiFSI at different molar ratios between the polymer and the salt. (c) Ionic conductivity comparison between poly­(DADMP)­FSI:LiFSI and poly­(DADMA)­FSI:LiFSI at 80 °C.

Figure 2c presents the ionic conductivity data of poly­(DADMP)­FSI:LiFSI at various poly­(IL):salt ratios at 80 °C and its comparison with data previously reported for ammonium poly­(DADMA).^16^ As with the poly­(DADMA)­FSI:LiFSI, a maximum in ionic conductivity is observed by varying the amount of LiFSI in the poly­(DADMP)­FSI:LiFSI SPEs. This composition-dependent behavior, where conductivity in poly­(IL)-in-salt electrolyte systems decreases significantly after a maximum value, has also been reported in previous works.^16,37,38^ This is ascribed to modifications in the ionic coordination environment, whereby there is a shift from predominantly poly­(IL)-FSI-Li cocoordination of the FSI anions toward an increasing fraction of Li-FSI-only coordination environments derived from the high salt content. Both SPEs reach the maximum ionic conductivity value at the ratio of 1:1.5, obtaining values at 80 °C of 1.5 × 10^–3^ S cm^–1^ for the phosphonium-based electrolyte and 9.5 × 10^–5^ S cm^–1^ for the ammonium-based. Noteworthy, the poly­(DADMP)­FSI: LiFSI SPE exhibits considerably higher ionic conductivity for all the ratios studied, suggesting that the intrinsic properties of the phosphorus heteroatom in the polymer backbone improve the ion transport, positively affecting the transport properties compared to the reference poly­(DADMA)-based electrolytes.

Figure 3a and b show the DSC data obtained for poly­(DADMP)­FSI:LiFSI and poly­(DADMP)­TFSI:LiFSI electrolytes. As observed previously for poly­(DADMA)­FSI or poly­(DADMA)­TFSI poly­(IL), the *T*_g_ value of the SPEs decreases upon the addition of the alkali metal salt. The addition of a salt disrupts the polycation–anion interactions, thereby acting as a plasticizer, which facilitates an increase in the polymer backbone dynamics, provoking a decrease in the *T*_g_. Poly­(DADMP)­FSI:LiFSI showed the lowest *T*_g_ of −58 °C at the poly­(IL):salt ratio of 1:1.5, which also corresponds to the composition with the highest ionic conductivity. Similarly, for the poly­(DADMP)­TFSI:LiFSI systems, the lowest *T*_g_ was observed for the composition giving the highest ion conductivity, with a *T*_g_ of −33 °C at the 1:2.5 composition. Further salt addition in both cases leads to the appearance of new endothermic peaks, specifically at ∼70 °C for the poly­(DADMP)­FSI: LiFSI 1:2 sample and at ∼0 and ∼60 °C for the poly­(DADMP)­TFSI:LiFSI 1:3 sample, related to the melting of a new crystalline phase formed between the poly­(IL)­s and the LiFSI salt. Indeed, previous DSC analysis of these higher salt concentration SPE systems has indicated the formation of new phases, distinct from the pure salt, with melting temperatures typically above 150 °C.^30,39^

**Figure 3:**
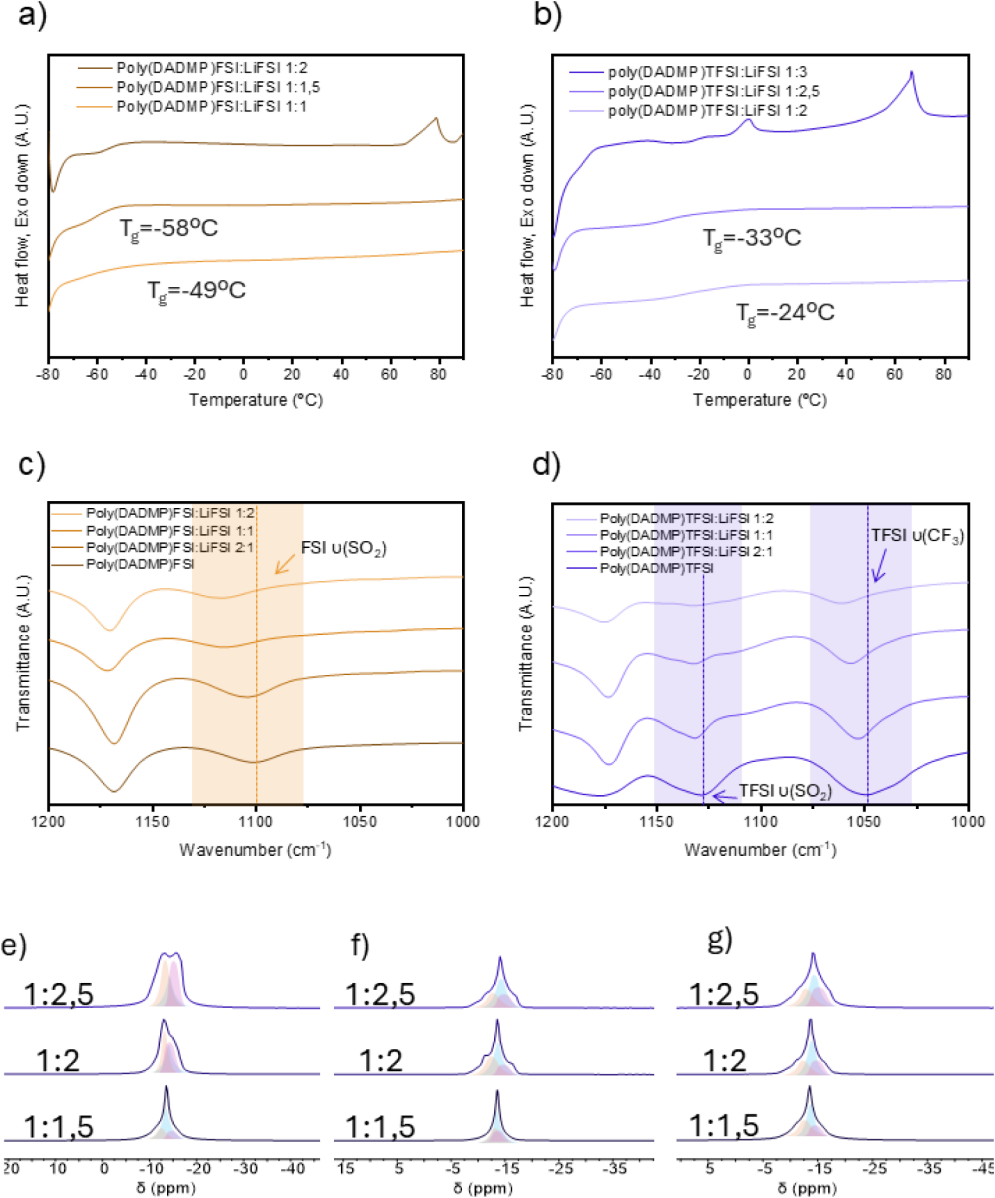
Characterization of poly­(IL)-in-salt SPEs: (a) and (b) DSC measurements of poly­(DADMP)­FSI:LiFSI and poly­(DADMP)­TFSI:LiFSI, respectively, at different molar ratios. (c,d) FTIR measurements of poly­(DADMP)­FSI:LiFSI and poly­(DADMP)­TFSI:LiFSI, respectively, at different molar ratios. (e), (f), and (g) Solid-state static NMR measurements of poly­(DADMP)­TFSI:LiFSI SPE at different compositions: 293 K without heating or cooling (e); measurements after heating to 333 K (f); and measurements at 293 K after cooling down (g).

It is also interesting that the mixed anions apparently have a higher LiFSI solubility, according to visual observations and the DSC traces. Despite the extended solubility range of LiFSI salt in poly­(DADMP)­TFSI with respect to poly­(DADMP)­FSI, the ion conductivities are lower across the whole range of temperatures investigated. This suggests that the entropic effect of mixing the anions works in favor of solubility, but at the same time, it does not translate into enhanced ion dynamics; instead, ion transport is reduced in the mixed system (TFSI-FSI), which is consistent with the higher *T*_g_ values displayed by poly­(DADMP)­TFSI:LiFSI SPEs compared with poly­(DADMP)­FSI:LiFSI. A lower *T*_g_ can certainly account for faster dynamics in the system; however, we note that at these high salt concentrations, the *T*_g_ reflects contributions from both the polymer backbone and the amorphous salt phase. It seems that the TFSI anion results in coordination environments between the polymer and lithium, which restrict dynamics in the entire system. The effective perturbation of the coordination environment of FSI^–^ and the establishment of polycation–anion interactions following the salt addition are further confirmed by FTIR spectroscopy. Full spectra are reported in Figure S14, while Figure 3c and d display the expanded spectra of the FSI–FSI and TFSI-FSI systems for the neat poly­(IL)­s and at different poly­(IL):salt compositions. The band assigned to the SO_2_ and CF_3_ stretching modes shifts to higher wavenumbers as the poly­(IL):salt ratio increases, indicating a modification of the coordination environment of the polycation counteranions. This blue shift is consistent with the enhanced coordination of FSI or TFSI anions in a cocoordinating environment involving polycation anions and Li^+^. In the polycation-FSI/TFSI-Li^+^ cocoordinating state, the increased electrostatic repulsion, which compresses the bonded anions, leads to a band shift toward higher vibrational frequency.

Interestingly, comparing the spectral shift of the poly­(DADMP)­FSI:LiFSI SPE with that of the poly­(DADMA)­FSI:LiFSI,^16^ the band at 1100 cm^–1^ is less shifted in the phosphonium-based electrolyte. This reduced shift suggests a weaker interaction or a lower degree of coordination between Li cations and FSI anions in the phosphonium-based system compared with the ammonium analogue, which may confer greater freedom of motion to the lithium ions within the SPE.

Multinuclear solid-state NMR is a powerful tool to interrogate both ion coordination and ion dynamics in solid-state electrolytes.^18,40,41^ ^7^Li NMR directly interrogates the lithium cation in these systems, so variable-temperature NMR was carried out to determine the effect of temperature on the relative dynamics of these species for different compositions. The static ^7^Li NMR for poly­(DADMP)­TFSI/LiFSI systems was investigated at 293 K and 333 K upon heating from a lower temperature up to 333 K (Figure 3e and f, respectively). Figure 3g shows the spectra at 293 K after cooling from the higher temperature excursion. Several interesting observations can be made here: first, all spectra present multiple peaks at room temperature (particularly prominent in spectra for 1:2 and 1:2.5), which change with temperature and composition. In the case of the 1:1.5 composition, a dominant peak occurs at 13 ppm, which also grows in intensity in the other two samples at higher temperatures and seems stable for at least the duration of the experiment on cooling down to 293 K once again (Figure 3g). This resonance is the narrowest peak and most likely represents the most mobile lithium species. Given the complex coordination environments suggested by previous work, both in the single-FSI system and in mixed anion systems,^11,18,42^ it is perhaps not surprising to see these multiple sites for ^7^Li in the present systems. A similar shoulder was observed in the case of the ^7^Li NMR spectra in the poly­(DADMA)­FSI:LiFSI system at the highest salt concentration^16^ and was assigned to a new LiFSI-rich crystalline complex, which involves both LiFSI aggregates and the polymer backbone coordination. What is interesting here is the evolution of these structures, with a predominance of the less negative chemical shift with increasing LiFSI concentration, favoring the LiFSI aggregates. The intermediate chemical shift possibly represents the cocoordination environment of the polycation–anion-Li and the most negative shift is suggestive of a TFSI-rich lithium coordination. As temperature increases, there will be a more rapid exchange between these environments, with the cocoordination being dominant. Such complex coordination environments were verified by MD simulations for previous poly­(DADMA) ammonium systems and will be discussed further in the next section. Figure S15 presents the deconvolution analyses of the solid-state NMR spectra along with the corresponding peak assignments.

### Molecular Simulations of the Ionic Structure and Dynamics in Phosphonium Poly­(IL)-In-Salt Solid Polymer Electrolytes

MD simulations provide more detailed insights into the structural environment and ion transport behavior. In particular, we tried to understand and rationalize the experimental observation that the SPEs based on the mixed-anion system exhibit lower ionic conductivity than the corresponding single-FSI system. Both systems were simulated at the same poly­(IL):salt ratio of 1:2 (Figure S16). The fast cooling simulation shows a higher *T*_g_ for the mixed-anion system in Figure 4b, consistent with the experimental observations. Because the salts are mixed well within the polymer matrix, the cation solvation shell in the mixed-anion system becomes hybrid in nature, with both FSI and TFSI present.

**Figure 4:**
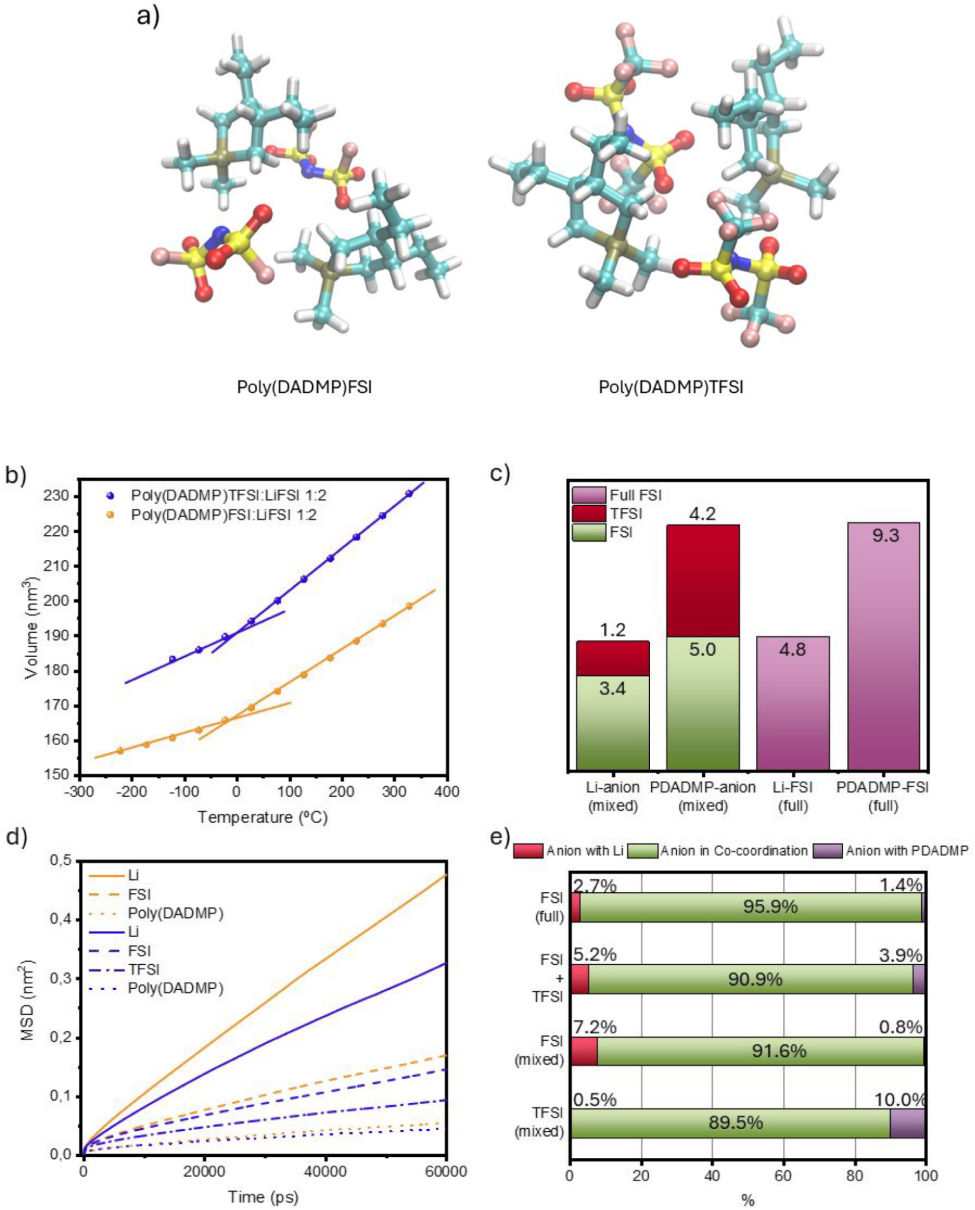
MD simulation results of the salt systems. (a) Poly­(DADMP)­FSI and poly­(DADMP)­TFSI simulated structures. (b) Volume-temperature plot of the poly­(DADMP)­FSI and poly­(DADMP)­TFSI with LiFSI salt at a 1:2 poly­(IL)-in-salt molar ratio. (c) Coordination number of FSI or TFSI in the first solvation shell of Li and poly­(DADMP) for the full and mixed anion systems. (d) MSD of Li, anions (through the N atom), and poly­(DADMP) (through the P atom) for the full and mixed anion systems. (e) Percentage of different anion coordination environments: anion coordinates only with Li (red), anion coordinates with both Li and poly­(DADMP) (green), and anion coordinates only with poly­(DADMP) (purple).

The total anion coordination number (CN) in the mixed-anion system remains comparable to that of the single-FSI system, with a minor reduction of 0.1–0.2 (Figure 4c). In the single-FSI system, an average of 5.1 FSI anions coordinates with Li, and 9.4 FSI anions coordinate with the poly­(DADMP) cation. These numbers decrease slightly to 4.9 and 9.3, respectively, in the mixed-anion system. Considering the 2:1 molar ratio of FSI to TFSI in the system, the average coordination of 3.6 FSI and 1.3 TFSI in the Li first coordination shell indicates a preferential coordination of Li toward FSI. In contrast, the presence of 4.2 TFSI and 5.1 FSI anions in the first coordination shell of poly­(DADMP) suggests its preference for TFSI. These results suggest a clear anion-coordination preference that we have seen in the mixed FSI/TFSI poly­(DADMA) system, where TFSI also preferentially associates with the polycation, while FSI favors coordination with Li.^34^

Ion diffusion was compared through mean-squared displacement (MSD) in Figure 4d. Higher diffusion is observed for both Li and the anions in the single-FSI system. In the mixed-anion system, TFSI clearly diffuses more slowly than FSI, which likely contributes to the overall reduction in ion mobility. We also analyzed three types of ion coordination states in Figure 4e: neat polycation–anion coordination, polycation–anion-Li cocoordination, and Li-anion coordination in a molten salt-like structure. In the single-FSI system, a high fraction of anions (95.9%) are in the cocoordination state, whereas in the mixed-anion system, this decreases to 90.9%. Instead, a growing molten salt region emerges, consisting of 7.6% FSI and 0.5% TFSI. In addition, 10% TFSI coordinates only with the polycation, further demonstrating its coordination preference. Therefore, the anions in the system experience a highly diverse coordination environment, as illustrated later through NMR experiments.

### Electrochemical Characterization and Performance of the Poly­(IL)-In-Salt SPEs in Li/Li Cells

Poly­(DADMP)­FSI:LiFSI 1:1.5 and poly­(DADMP)­TFSI:LiFSI 1:2 SPEs were selected for further electrochemical studies as they displayed the highest ionic conductivity while retaining longer-term phase homogeneity, as reported in Figure 5. First, cyclic voltammetry (CV) was performed by scanning the anodic and cathodic potentials using aluminum carbon-coated and copper working electrodes, respectively, and lithium metal as the counter and reference electrode, at 40 °C (Figure 5a and b). As depicted in Figure 5a, the cathodic scan from OCV poly­(DADMP)­FSI:LiFSI 1:1.5 shows a reduction peak centered at ∼2.2 V, attributed to the reduction of FSI anions,^43^ followed by a rapid increase in cathodic current density at −0.05 V vs Li^+^/Li assigned to the lithium plating on the copper electrode. On the other hand, the anodic scan is characterized by the absence of any significant faradaic reactions in the range between OCV and 4.5 V vs Li^+^/Li. Even at higher potential, the electrolyte is stable, and only an extremely limited current density increase was observed at 5 V vs Li^+^/Li. Regarding the poly­(DADMP)­TFSI:LiFSI 1:2 SPE (Figure 5b), the cathodic scan is characterized by a broader reduction peak centered at ∼1.4 V vs Li^+^/Li; this is ascribed to the irreversible reduction of TFSI anions and SEI formation. A reversible plating and stripping of lithium is observed between −0.5 V and 0.5 V vs Li^+^/Li;^44^ meanwhile, the anodic scan in this sample is similar to that observed for the poly­(DADMP)­FSI:LiFSI 1:1.5.

**Figure 5:**
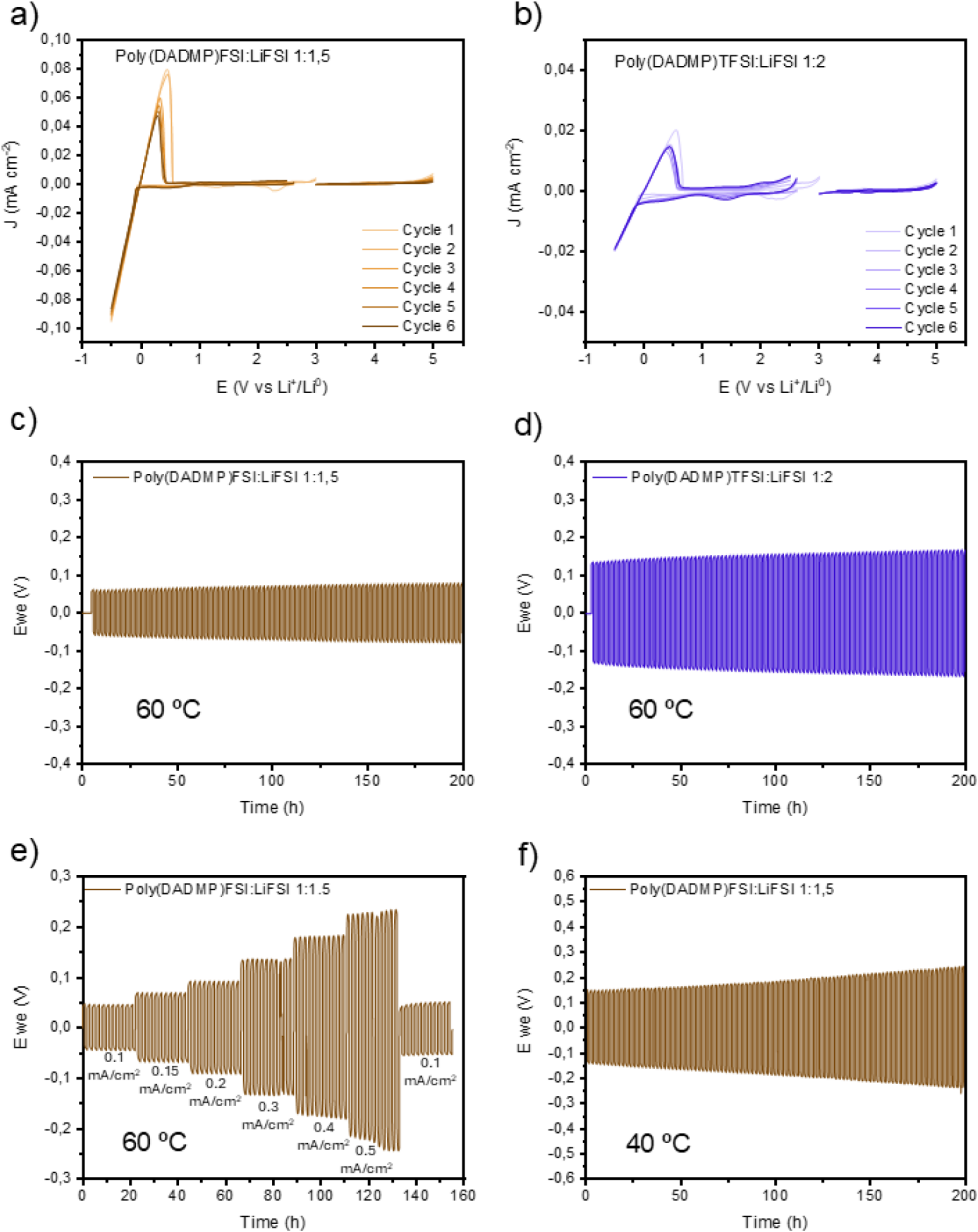
(a) Cyclic voltammetry of the FSI-FSI electrolyte on Cu (cathodic) and Al carbon-coated (anodic); (b) cyclic voltammetry of the TFSI-FSI electrolyte on Cu (cathodic) and Al carbon-coated (anodic); (c) Li|Li symmetric cycling at 60 °C of the FSI-FSI electrolyte; (d) Li|Li symmetric cycling at 60 °C of the TFSI-FSI electrolyte; (e) Li|Li symmetric cycling at 40 °C of the FSI-FSI electrolyte; (f) C-rate experiment of the FSI-FSI electrolyte.

Noteworthy, the single-FSI SPE shows higher current densities related to plating/stripping processes, as well as a more defined and sharp change in the current density at the onset of Li^+^ plating, which starts at −0.05 V vs Li^+^/Li and remains almost constant over cycling, as illustrated in Figure S17. By contrast, the mixed TFSI-FSI SPE material is characterized by lower current densities and a non-negligible resistive contribution during the plating, which starts at −0.08 V vs Li^+^/Li and decreases over cycling up to −0.12 V vs Li^+^/Li, suggesting the formation of a less stable and more resistive SEI. The CV profiles obtained for the single-FSI sample indicate a less resistive and more uniform Li^+^ plating and stripping process, reflecting more favorable redox kinetics, low interfacial resistance, and stable and highly conductive SEI formation. This behavior is intrinsically connected to the higher ionic conductivity observed in the single-FSI SPE case versus the mixed TFSI-FSI system.

Lithium transference number ( $t_{Li^{+}}$ *t*) of both SPEs has been calculated through the ratio between the steady-state current (*I*_ss_) at *t* = 10 h and the initial one at *t* = 0 (*I*_0_) in a Li|Li symmetric cell that was subjected to a 50 mV polarization bias (Δ*V*) (Figure S18). The data indicate a $t_{Li^{+}}$ *t* of 0.6 for the poly­(DADMP)­FSI:LiFSI 1:1.5 SPE and 0.7 for the poly­(DADMP)­TFSI:LiFSI 1:2 SPE. It is worth noting that the $t_{Li^{+}}$ calculated for both poly­(IL) SPEs is significantly higher when compared to the PEO-based SPEs, which rarely exceed a value of 0.3.^45,46^ Indeed, the obtained $t_{Li^{+}}$ values for the phosphonium-based poly­(IL)­s SPEs are higher or comparable with other ammonium poly­(IL)­s-in-salt systems previously investigated, where the values do not overcome 0.67 with measurements run at higher temperatures (80 °C).^16,37^

To further evaluate the electrochemical features of the novel phosphonium poly­(IL) SPEs for future implementation in energy storage devices, Li|Li symmetric cells were assembled with both single-FSI and mixed TFSI-FSI SPEs to conduct extended cycling tests at 0.1 mA cm^–2^, at 60 °C (Figure 5c and d). During the constant current density plating and stripping measurement, poly­(DADMP)­FSI:LiFSI 1:1.5 exhibited a significantly lower overpotential of ∼0.06 V compared to the ∼0.14 V observed for the poly­(DADMP)­TFSI:LiFSI 1:2 sample, as well as a reduced overpotential increase after 200 cycles at 60 °C, specifically 0.01 V for the single-FSI SPE and 0.04 V for the TFSI-FSI mixed one. The outcomes are consistent with the behavior observed during the CV experiments, corroborating the lower ion mobility of the FSI–TFSI mixed SPE.

Following the promising stability and cycling performance observed in Li∥Li symmetric cells, we further challenged the system to assess its tolerance under more demanding operating conditions. Two more experiments were performed. First, the Li cells were cycled at different current densities (0.1, 0.15, 0.2, 0.3, 0.4, and 0.5 mA cm^–2^), 10 cycles each and then cycling again at 0.1 mA cm^–2^ to evaluate the overpotential retention. Second, the Li cells were cycled at 0.1 mA cm^–2^ at 40 °C to mimic operating conditions closer to room temperature applications. The results of these two experiments are shown in Figure 5e and f for Poly­(DADMP)­FSI:LiFSI 1:1.5 and in Figure S19 for poly­(DADMP)­TFSI:LiFSI 1:2.

Poly­(DADMP)­FSI:LiFSI 1:1.5 displays stable cycling across the whole range of current densities with a gradual overpotential increase of up to ∼0.23 V at 0.5 mA cm^–2^. Indeed, after cycling at higher current densities, the cell quickly recovers the initial overpotential when cycled back at 0.1 mA cm^–2^, suggesting high stability in contact with the Li metal electrode and excellent retention of electrochemical performance. On the other hand, despite the limited and stable overpotential at 0.1 and 0.15 mA cm^–2^, poly­(DADMP)­TFSI:LiFSI 1:2 undergoes a short-circuit when moving to 0.2 mA cm^–2^. This result is likely correlated to the formation of an unstable interface between the polymer electrolyte and the Li metal electrode, arising from suboptimal interfacial engineering. It is worth noting that poly­(DADMP)­FSI:LiFSI 1:1.5 showed improved performance compared with similar previously investigated sSPEs.^16,37^ Namely, it substantially outperforms the ammonium-based poly­(DADMA)­FSI:LiFSI 1:1.5 in terms of overpotential at 0.2 mA cm^–2^, while showing comparable values to the protic ammonium-based poly­(DAMAH)­FSI:LiFSI 1:2, but with superior stability during cycling.

Aiming to demonstrate the feasible application of the phosphonium-based SSPEs in real applications, the samples are cycled at 0.1 mA cm^–2^ at 40 °C. Both systems exhibit a limited overpotential that slightly increases over cycling, reaching 0.25 V for poly­(DADMP)­FSI:LiFSI 1:1.5 (Figure 5f) and 0.77 V for poly­(DADMP)­TFSI:LiFSI 1:2 (Figure S19). Comparing both systems, the poly­(DADMP)­FSI:LiFSI 1:1.5 demonstrates more stable cycling at 40 °C. A summary of the main electrochemical results is provided in Supplementary Table 4 of Supporting Information.

The overall result of the electrochemical characterization and performance of the solid electrolytes investigated indicates that poly­(DADMP)­FSI:LiFSI 1:1.5 has excellent electrochemical properties, good stability, and outstanding performance in Li|Li symmetric cells, with superior characteristics compared to SPEs previously studied, contributing to the development of next-generation energy storage devices.

## Conclusions

In this work, a novel class of solid polymer electrolytes for fast lithium-ion conduction based on phosphonium poly­(ionic liquid)­s was presented. Two new poly­(diallyldimethylphosphonium) poly­(IL)­s, namely poly­(DADMP)­FSI and poly­(DADMP)­TFSI, were successfully synthesized by cyclopolymerization of the diallyldimethylphosphonium monomer, and after ion exchange to substitute the halide anion. The characterization techniques employed, such as NMR or FTIR, confirm the formation of a 5-membered ring polymer backbone. This new polymer can be meaningfully compared with the ammonium poly­(DADMA) poly­(IL)­s due to its similar structure, as they differ only in the heteroatom. Moreover, characterization techniques have shown that phosphonium poly­(DADMP) exhibits superior mechanical properties, such as a lower glass transition temperature than poly­(DADMA) (40 °C vs 116 °C) in the case of polymers bearing TFSI as the counteranion, as well as reasonably high ionic conductivities of 2.6 × 10^–6^ S cm^–1^ at 100 °C in the neat polymer.

The nature of the coordination environments and ionic interactions of the SPEs obtained by mixing poly­(DADMP)­FSI and poly­(DADMP)­TFSI with different contents of lithium fluorosulfonamide (LiFSI) was deeply investigated by FTIR, DSC, ionic conductivity, solid-state Li NMR, and molecular modeling simulations. Indeed, the phosphonium-based poly­(IL)­s-in-salt SPEs showed excellent electrochemical features, with the sample poly­(DADMP)­FSI:LiFSI 1:1.5 achieving ionic conductivity values of 1.5 × 10^–3^ S cm^–1^ at 80 °C and 4.6 × 10^–4^ S cm^–1^ at 50 °C, with elevated $t_{Li^{+}}$ of 0.7. poly­(DADMP)­TFSI:LiFSI systems exhibited superior Li salt solubility, reaching the highest ionic conductivity of 5.3 × 10^–4^ S cm^–1^ at 80 °C with the poly­(DADMP)­TFSI:LiFSI 1:2.5 sample. At extended storage times, this sample develops a crystalline component that could affect the electrochemical performance of the electrolyte. For this reason, poly­(DADMP)­TFSI:LiFSI 1:2 was chosen for further electrochemical characterization due to its phase stability (i.e., absence of an obvious crystalline component), showing high $t_{Li^{+}}$ values of up to 0.6. Both SPEs were characterized by a broad electrochemical stability window, with the anodic limit overcoming 4.5 V vs Li^+^/Li. The excellent electrochemical properties of the SPEs, along with the peculiar ion-coordinating ability of phosphonium-based SPEs, allowed us to demonstrate lithium plating/stripping at 0.1 mA cm^–2^ in Li/Li symmetrical cell at 60 °C and 40 °C, reaching limited overpotentials of 0.06 V and 0.25 V, respectively. Furthermore, the use of poly­(DADMP)­FSI:LiFSI 1:1.5 SPE supported lithium electrochemistry without short circuits up to a current density of 0.5 mA cm^‑2^ with overpotentials less than 0.23 V at 60 °C, paving the way for future investigation and implementation in Li-metal and Li-ion battery cells.

## Supplementary Material



## Notes

Additional experimental details, including methods, materials, procedures, characterization data, NMR spectra, and computational details (PDF)
