# Supplementary material for: Phosphonium Poly(Ionic Liquid) Electrolytes for Fast Lithium-Ion Conduction
Source: J Am Chem Soc. 2026 Apr 29;148(18):19074–86. doi: 10.1021/jacs.6c02428 (PMC13184986; doi:10.1021/jacs.6c02428)
Supplement: Supplementary file 1 [file ja6c02428_si_001.pdf]

Supporting Information

# Phosphonium Poly(Ionic liquid) Electrolytes for Fast Lithium-Ion Conduction

Alejandro Herranz Berzosa[a,†], Kewei Cai[b,†], Gabriele Lingua[a], Daniele Mantione[a,d], Iñaki Santos[a], Federica Santino[a], Maria Forsyth[a,b,c,],Fangfang Chen[b*] and David Mecerreyes[a,c*]

[a] *POLYMAT, University of the Basque Country UPV/EHU, Avenida Tolosa 72, Donostia-San Sebastian 20018, Spain*

[b] *Institute for Frontier Materials, Deakin University, Burwood, Victoria 3125, Australia*

[c] *IKERBASQUE, Basque Foundation for Science, Bilbao 48011, Spain.*

*E-mail: david.mecerreyes@ehu.eus; fangfang.chen@deakin.edu.au

## METHODS

### Materials

Phosphorous trichloride ($PCl_3$) reagentPlus, 99%, Fe (powder) >99.9%, reduced, powder(fine), and Allylmagnesium chloride (2M in THF) were purchased in Sigma Aldrich. Aluminum trichloride ($AlCl_3$), 98.5%, the extra pure, anhydrous, powder was acquired in Acros Organics. Iodomethane ($CH_3I$), 99.5%, stab. with copper was purchased in Thermo scientific. Potassium chloride (KCl) for analysis was purchased in Panreac. Tetrahydrofuran (THF), 99.5%, extra dry over molecular sieves, stabilized obtained from Thermo Scientific. 2,2′-azobis(2-methylpropionamidine) dihydrochloride (AIBA, 98%) were purchased from Thermo Fisher. Lithium bis(fluorosulfonyl)imide (LiFSI, 99%) and lithium bis(trifluoromethanesulfonyl)imide (LiTFSI, 99%) were obtained from Solvionic.

### Characterization methods

**Nuclear Magnetic Resonance (NMR).** NMR spectra of the new materials in solution state were recorded using a Bruker Avance DPX 300 spectrometer operating at a resonance frequency of 75.5 MHz. Dimethyl sulfoxide (DMSO-d6) was used as a solvent at room temperature. $^7Li$ solid-state NMR experiments were performed with 11.7 T wide bore NMR spectrometer equipped with a Bruker AVANCE NEO console and PH MASDVT500W BL3.2 N-P/H double resonance probe. The material was packed into a 3.2mm rotor. The experiments were realised in static, the recycle delay was 5s and 256 scans were used. Larmor frequency was 195.25 MHz for 7Li.

**Fourier Transform Infrared Spectroscopy (FTIR).** FTIR spectras were recorded on a Bruker α II spectrophotometer equipped with a platinum ATR module featuring a diamond window.

**Thermogravimetric Analysis (TGA).** The thermal stability was evaluated by TGA using a TGA Q500 instrument from TA Instruments. Samples were heated at a rate of 10 °C/min under a nitrogen atmosphere from room temperature to 800 °C.

**Differential Scanning Calorimetry (DSC).** The glass transition temperatures (Tg) were determined by DSC using a DSC Q2000 apparatus from TA Instruments. Scans were performed at heating and cooling rates of 10 °C/min from −80 to 200 °C.

**Gel permeation chromatography (GPC).** Size-exclusion chromatography was performed in THF as the mobile phase at a flow rate of 1.0 $mL\cdot min^{-1}$, using an LC-20AD (Shimadzu) pump coupled to a Waters 717 plus autosampler and a Waters 2414 refractive index detector. Separation was achieved using a Styragel guard column followed by three Styragel columns in series (HR1, HR2, and HR2; Waters) thermostatted at 35 °C.

**Electrochemical Characterization.** Electrochemical impedance spectroscopy (EIS) was employed to determine the ionic conductivity of the poly(IL)s-salt samples in an Autolab 302 N potentiostat galvanostat at different temperatures (30–100 °C) with an equilibration time of 20 min at each temperature before measurement to ensure equilibration. The samples were placed between two stainless steel electrodes (surface area of 0.5 $cm^2$) and the thickness was kept constant using a 500 μm silicon spacer. The measurements were obtained in the

range between 300 kHz and 1 Hz, with a perturbation amplitude of 20 mV. The cell assembly was carried out inside a glovebox to avoid any humidity uptake effect, which may alter the ionic conductivity.

The ohmic resistance of the polymer electrolyte sample, obtained from the Nyquist plot at the low-frequency end of the semicircle, was used to calculate the ionic conductivity using the following equation 1.

$$\sigma = d/(A \times R) \qquad \textbf{Equation 1}$$

Where σ is the ionic conductivity, A is the area of the electrolyte in contact with the electrode and R is the Ohmic resistance.

Cyclic voltammetry (CV) was performed using a VMP3 potentiostat (Bio-Logic Science Instruments) to determine the electrochemical stability window (ESW) of the polymer electrolyte at 40 °C. The two-electrode cell setup was used with Li metal as counter and reference electrodes, with copper and Al-carbon coated substrates used as working electrodes to investigate the cathodic and anodic stability, respectively. The potential sweeps were carried out between the OCV and 5 V vs $Li^+/Li$ for the anodic scan and −0.5 V vs $Li^+/Li$ for the cathodic scan, both at a constant rate of 1 mV $s^{-1}$. The CV was conducted in a coin cell configuration employing an O-ring separator with an internal diameter of 10 mm and 500 μm thickness.

The lithium-ion transport number ($t_{Li^+}$) was determined at 60 ˚C in a symmetric Li/SSPE/Li cell, which was subjected to a 50 mV polarization bias ($\Delta V$). An O-ring separator with an internal diameter of 10 mm and 500 μm thickness was used. The initial current at $t$ = 0 ($I_o$) and at steady state ($I_{ss}$) at $t$ = 10 h were recorded. The $t_{Li+}$ was calculated by using the ratio between these values.

Lithium plating and stripping analysis were conducted in Li metal symmetrical cell configuration at different current densities (0.1, 0.15, 0.2, 0.3, 0.4, 0.5 mA $cm^{-2}$) at 60 ˚C while the long cycling at 0.1 mA $cm^{-2}$ was conducted at 60 and 40 °C. Electrochemical characterization was conducted using coin cells CR2032 assembled inside the Ar-filled glovebox ($H_2O$ and $O_2$ content <0.1 ppm) to avoid moisture contamination. We note that the materials examined here were very soft and so a spacer was used to maintain 500 μm separation between the two Li electrodes.

## EXPERIMENTAL

### Synthesis of Diallyldimethylphosphonium monomer (DADMP)

In a controlled argon atmosphere, phosphorus trichloride (1.5 mol) was combined with aluminium trichloride (1.7 mol) and heated to 70 °C. Subsequently, the reaction mixture was cooled to -15 °C using a sodium chloride/ice bath, and Iodomethane was added dropwise, maintaining continuous stirring. The mixture was kept stirring in the ice bath for 60 minutes, and 60 additional minutes at room temperature. During this process, the initial yellow liquid transitioned to an orange solid compound. In the subsequent step, potassium chloride (1.7 mol) and Fe powder (90 g) were introduced into the reaction mixture, which was then heated to 260 °C. Under these conditions, a distilled mixture of iodine and methyl phosphorus was obtained. Once we have the methylphosphorous dichloride, the synthesis of diallyldimethylphosphonium monomer has been performed. A 2-neck round-bottom flask was filled with 1 eq. of Methyl phosphorus dichloride previously synthesised

(1.8 ml, 160 µl) and dissolved in 3 ml of dry THF under an argon atmosphere. The solution was allowed to reach -78˚C in an ethanol/$N_2$ liquid bath, and 2 eq. of allyl magnesium bromide (solution 1.0M in THF) were slowly added. The solution was allowed to reach room temperature and stirred overnight. The day after, a solid was formed, and the liquid part was filtered from the solid with 140 µm PFTE filters and transferred to an argon-filled round-bottomed flask equipped with a stirrer. The round-bottomed flask was cooled down to -15 °C with a NaCl/iced bath, and 1.5 eq of CH3I was slowly added. The reaction was allowed to reach room temperature again and stirred for 24 hours. After the appropriate reaction time, a solid precipitate formed in the round-bottom flask. The liquid is removed by decantation. The solvent from the liquid part is removed under vacuum and a yellowish solid is obtained.$^{1}$H NMR (400 MHz, DMSO) δ 5.80 (ddtd, *J* = 17.5, 10.3, 7.6, 5.2 Hz, 0H), 5.42 (m, *J* = 5.5, 1.5 Hz, 0H), 3.33 – 3.15 (m, 1H), 1.92 – 1.78 (m, 1H).$^{13}$C NMR (101 MHz, DMSO) δ 125.20 (dd, *J* = 22.3, 10.0 Hz), 123.45 (dd, *J* = 24.9, 12.4 Hz), 26.36 (d, *J* = 49.2 Hz), 5.03 (d, *J* = 53.1 Hz).$^{31}$P NMR (162 MHz, DMSO) δ 28.63.

**Synthesis of poly(diallyldimethylphosphonium)FSI and TFSI polymers (poly(DADMP)FSI and poly(DADMP)TFSI)**

1.0 g of diallyldimethylphosphonium iodide monomer was dissolved in 10 ml of water (10 wt%), while 0,05 g of the initiator, 2,2'-Azobis(2-methylpropionamidine) dihydrochloride (AIBA), was dissolved in the minimum necessary amount of water. Both solutions were degassed with argon for 20 minutes, and the monomer solution was preheated to 60 °C. Once degassing was complete and the monomer solution reached 60 °C, the initiator solution was injected into the monomer solution using a syringe. The reaction mixture was then heated to 70 °C and allowed to react overnight. The following day, a red precipitate formed in the round-bottom flask. The precipitate was separated by centrifugation, and the supernatant was decanted. The polymer was recovered from the liquid phase by precipitation in acetone. After decantation of the acetone, the polymer was redissolved in water, and an aqueous solution of Lithium bis(fluorosulfonyl)imide (LiFSI) (1.2 eq.) was added dropwise, leading to the formation of a precipitate. The precipitate was collected by centrifugation and decantation, then dried under vacuum until a white powder was obtained.$^{1}$H NMR (400 MHz, DMSO) δ 2.45 – 2.15 (m, 1H), 2.12 – 1.73 (m, 31H), 1.29 – 1.18 (m, 7H), 1.19 – 0.72 (m, 1H).$^{13}$C NMR (126 MHz, DMSO) δ 48.99 – 44.09 (m), 28.54, 26.72 – 22.14 (m), 8.35 (dd, *J* = 79.7, 47.3 Hz), 5.86 – 2.92 (m).$^{31}$P NMR (202 MHz, DMSO) δ 57.46 – 11.71 (m). The synthesis of poly(DADMP)TFSI followed the same procedure, changing the salt used for the ion-exchange step. Lithium bis(trifluoromethanesulfonyl)imide (LiTFSI) (1.2 eq.) was added dropwise to the polymer solution in water, leading to the formation of another precipitate. That precipitate is also collected by centrifugation and dried under vacuum after decantation of the liquid part. $^{1}$H NMR (400 MHz, DMSO) δ 2.45 – 2.15 (m, 1H), 2.12 – 1.73 (m, 31H), 1.29 – 1.18 (m, 7H), 1.19 – 0.72 (m, 1H).$^{13}$C NMR (101 MHz, DMSO) δ 119.54 (q, *J* = 321.8 Hz), 28.47, 24.45, 9.44 – 6.45 (m), 4.74 (d, *J* = 51.6 Hz).$^{31}$P NMR (162 MHz, DMSO) δ 49.34 – 17.07 (m).

Diallyldimethylphosphonium monomer was characterized by $^{1}$H-NMR,$^{13}$C-NMR and $^{31}$P-NMR, and the spectra are shown in figure S1. The NMR sample has been prepared under a N2 flow to avoid contact with air due to the probability of the monomer reacting with the atmosphere.

$^1$H-NMR shows 1 signal in 1.92 ppm assigned to the methyl groups of the monomer. A multiplet at 3.33 ppm was observed and attributed to methylene protons and two signals at 5.42 and 5,8 ppm were assigned to the vinylic protons and allylic methylene protons, respectively.

In $^{13}$C-NMR, a signal at 5.03 ppm is assigned to the methyl groups and a signal at 26.36 ppm corresponds to the methylene carbons of the monomer. Both resonances appear as doublets due to $^{13}$C-$^{31}$P coupling, consistent with their position adjacent to the phosphorous atom in the monomer. The spectra also show two signals at 123.45 and 125.20 ppm, respectively assigned to the vinylic protons.

$^{31}$P-NMR spectra show a singlet signal at 28.63 ppm. Phosphor-based starting materials are very reactive and air sensitive to oxidation. The fact of only having one signal in the 31P-NMR suggests no oxidations or undesired side reactions during the synthesis procedure.

The NMR data give enough evidence to determine that the synthesis of the diallyldimethylphosphonium monomer was successful, and a pure monomer was obtained.

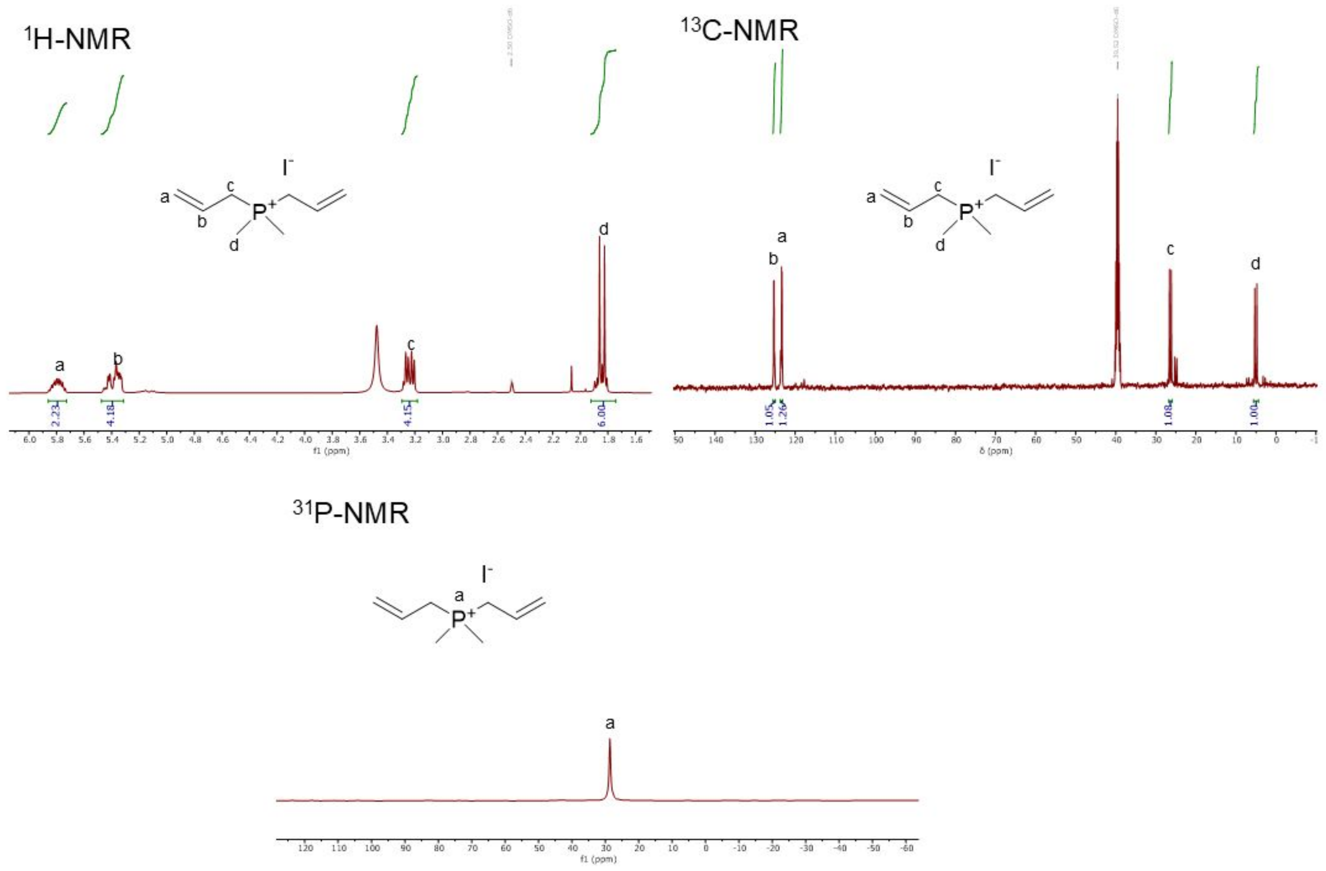


***Figure S1***: *$^{1}$H-NMR(top left), $^{13}$C-NMR(top right) and $^{31}$P-NMR (bottom) spectras of diallyldimethyl phosphonium monomer.*

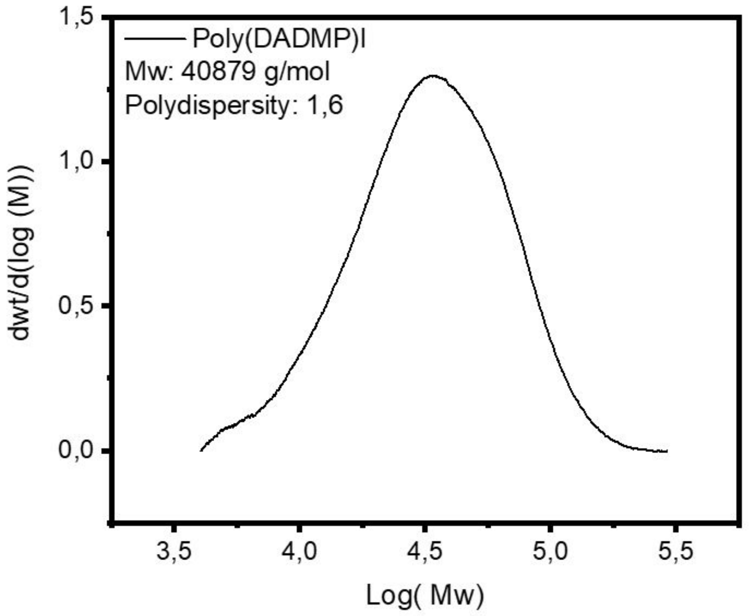


***Figure S2:*** *Molecular weight of Poly(DADMP)I calculated by Gel Permeation chromatography.*

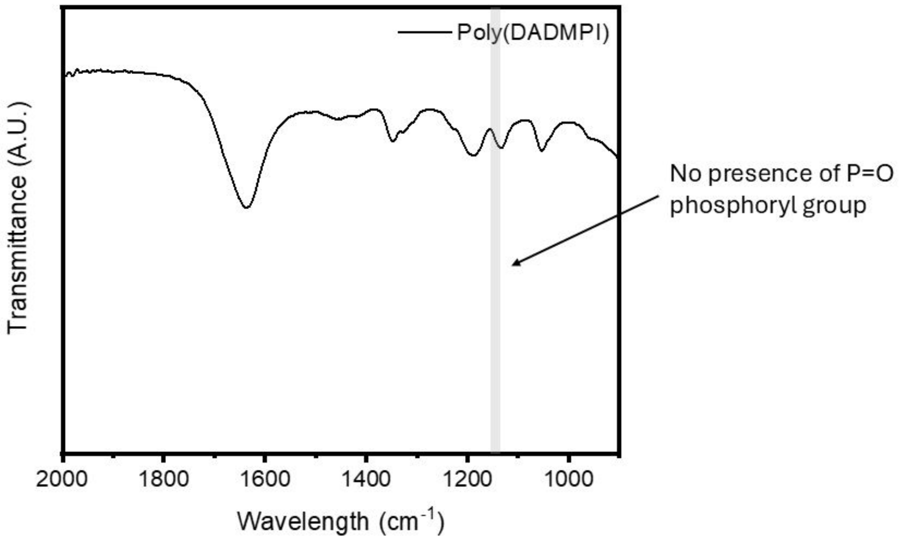


*Figure S3: FTIR of Poly(DADMP)I.*

***Supplementary Table 3**: Solubility table summarize of Poly(DADMP)FSI and Poly(DADMP)TFSI*

| **Solvent** | **Poly(DADMP)FSI** | **Poly(DADMP)TFSI** |
|---|---|---|
| THF | Partial solubility | Partial solubility |
| ACN | Soluble | Soluble |
| DCM | Partial solubility | Partial solubility |
| DMF | Partial solubility | Partial solubility |
| DMSO | Soluble | Soluble |
| NMP | Soluble | Soluble |
| PC | Partial solubility | Partial solubility |
| H2O | Not soluble | Not soluble |
| MeOH | Low-moderate solubility | Low-moderate solubility |
| Hexane | Not soluble | Not soluble |

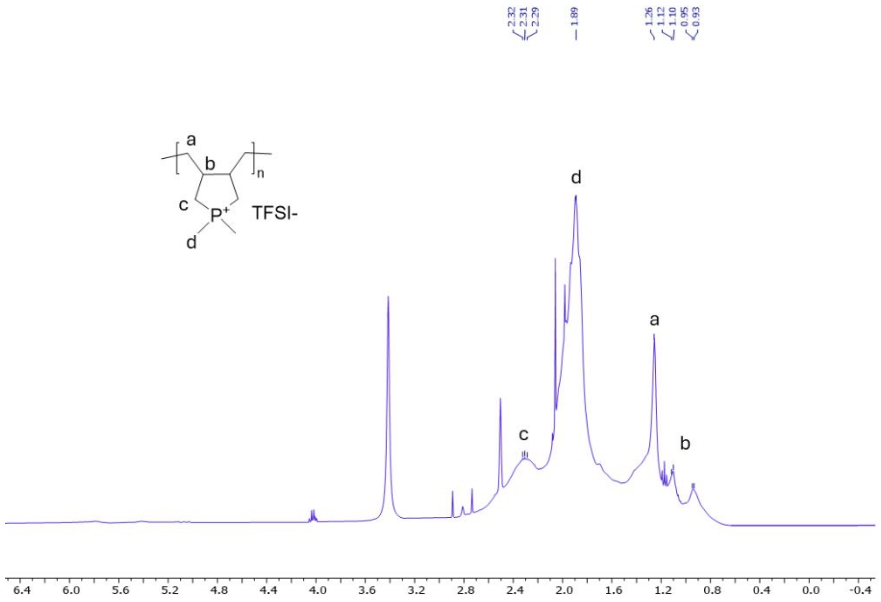


***Figure S4**: $^{1}$H NMR of Poly(Diallyldimethyl phosphonium)TFSI (poly(DADMP)TFSI*

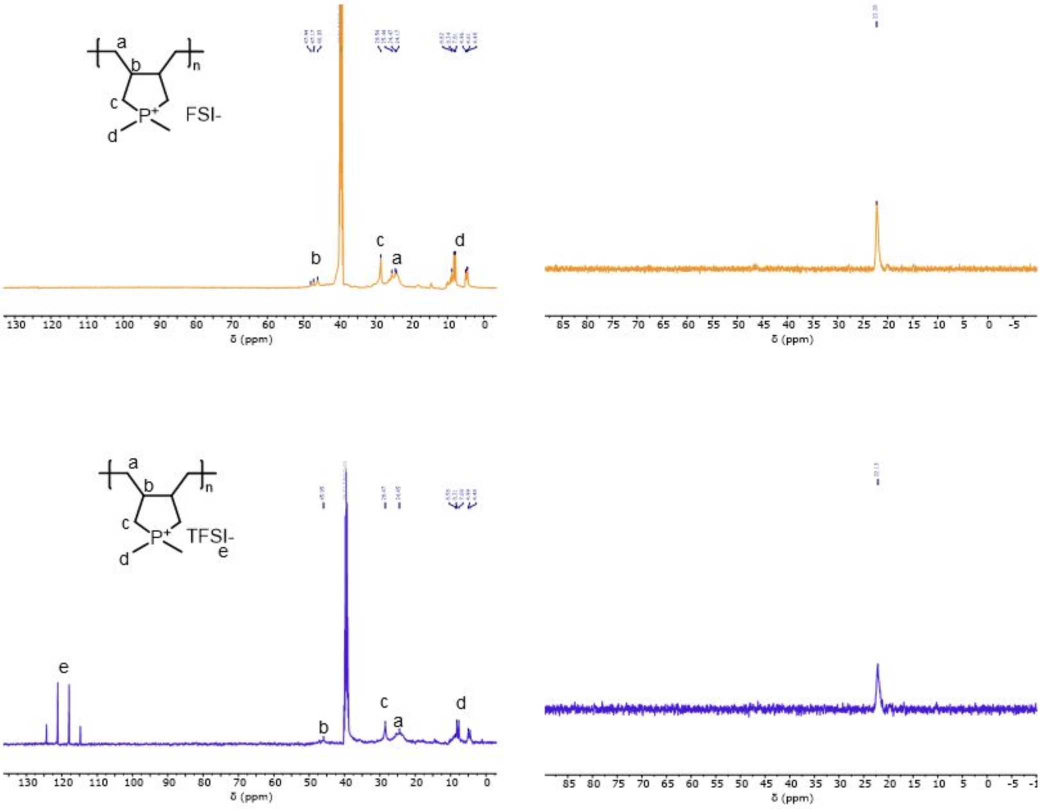


*Figure S5*: *$^{13}$C-NMR(left) and $^{31}$P-NMR (right) of poly(DADMP)FSI(top) and poly(DADMP)TFSI(bottom).*

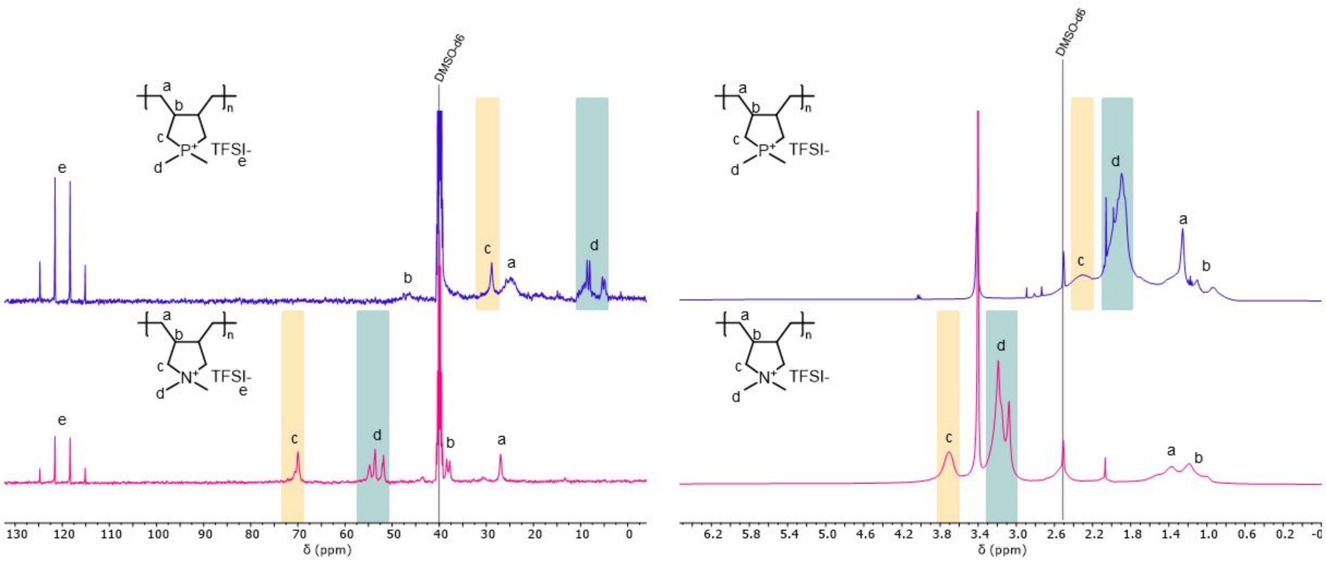


*Figure S6: Comparison of the NMR spectras of $^{13}C$ NMR (left) and $^{1}H$ NMR (right) of Poly(DADMP)TFSI (top) and Poly(DADMA)TFSI(bottom).*

## ***Partiall oxidation of the polymer.***

The polymer shows partial oxidation when is exposed to oxygen during long periods, changing the appearance from a white powder to a yellowish. The oxidation its been confirmed through 31P-NMR experiments at the oxidazed samples, showing 2 signals. The appearance of a new signal at 47 ppm indicates the partial oxidation of the polymers. Figure below shows the physical appearance of the polymer inside the glovebox and after 2 weeks of exposing into the atmosphere. The polymer goes from white to yellow. Both polymer (Poly(DADMP)FSI and Poly(DADMP)TFSI) shows the same trend. After this colour change, 31P-NMR is performed to both polymers. The results show different signals, one at 22 ppm providing from the phosphonium atom in the polymer and others at around 40 to 50 ppm assigned to the oxidated form of the polymers, indicating partial oxidation of the polymers.

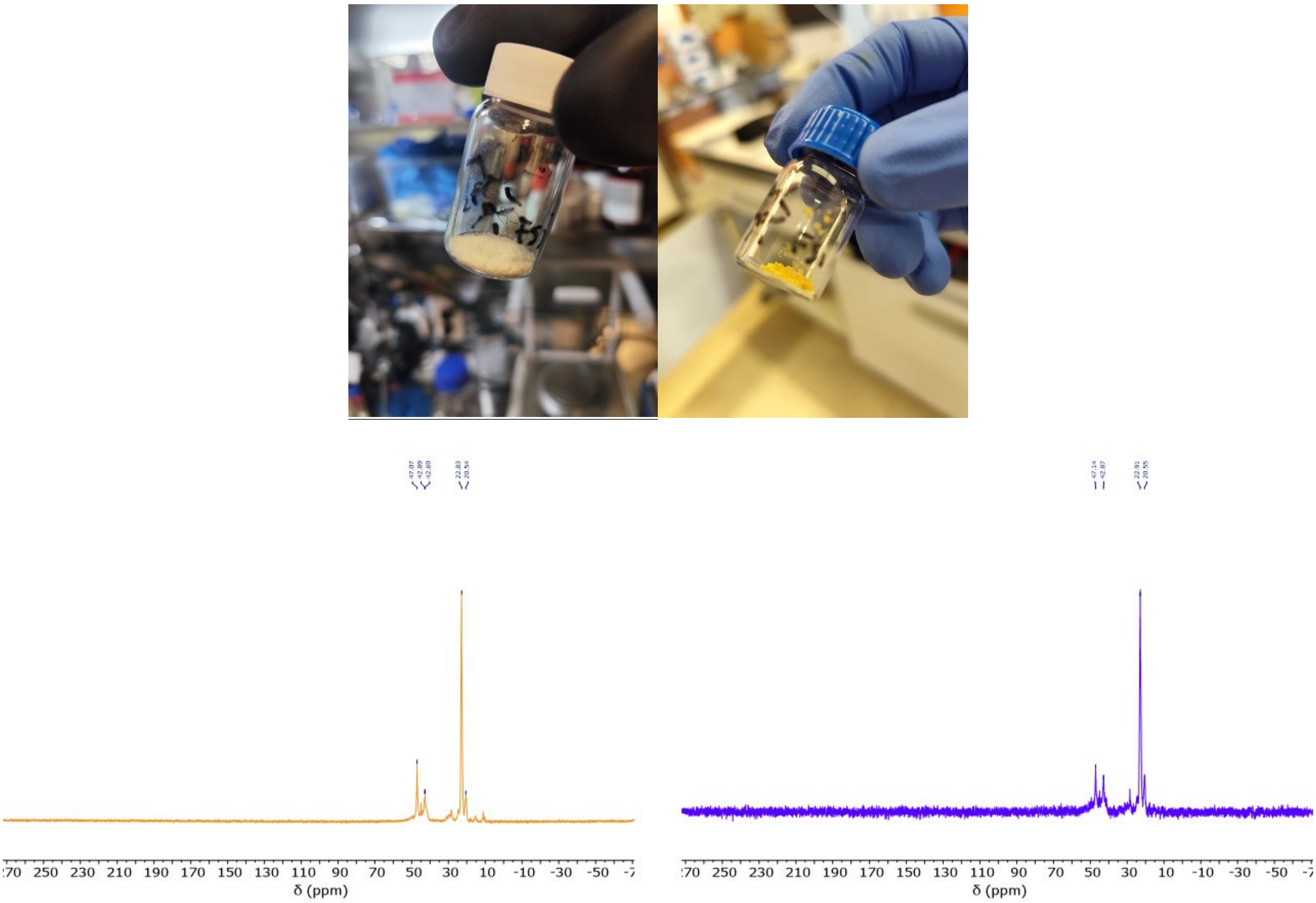


***Figure S7**: Physical apearence of poly(DADMP)FSI without the presence of oxygen (top left) and in presence of oxygen for extended periods of time (top right) and 31P-NMR of poly(DADMP)FSI (bottom left) and poly(DADMP)TFSI (bottom right) after exposition of oxygen.*

The HSQC spectras reveal direct one-bond correlations between protons and their attached carbons. Notable cross-peaks include the $CH_2$ group at δ H 1.25 ppm / δ C 25 ppm (cross-peak A), the methine CH at δ H 1.1 ppm / δ C 50 ppm (cross-peak B), the methylene adjacent to the heteroatom at δ H 2.1 ppm / δ C 30 ppm (cross-peak C), and the methyl groups at δ H 1.9 ppm / δ C 5–8 ppm (cross-peaks D).

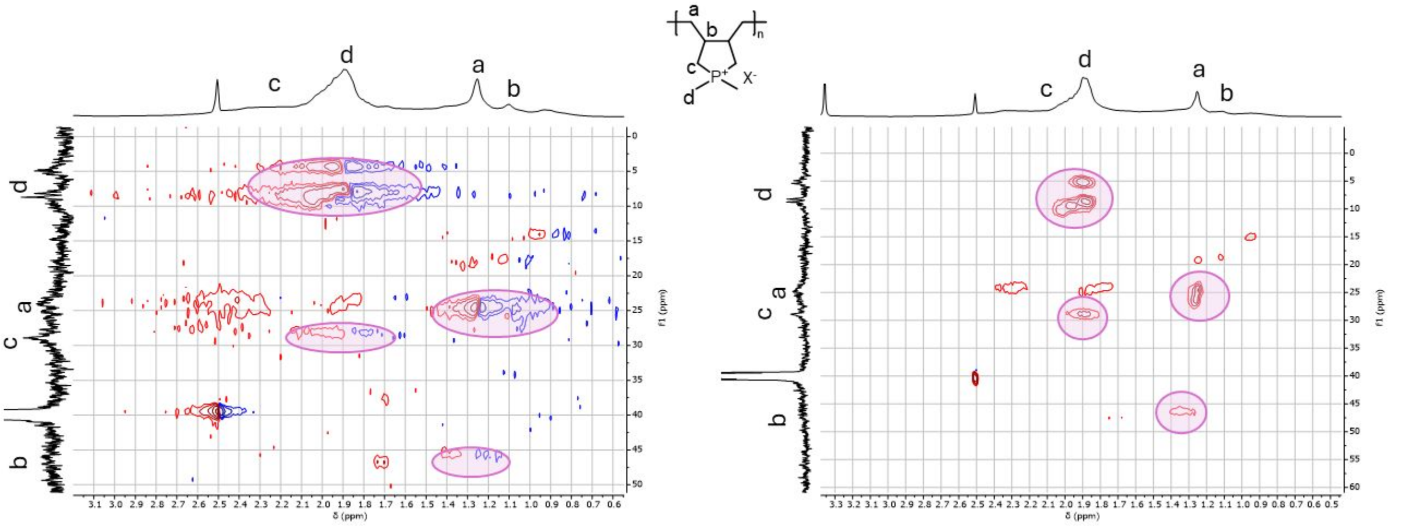


***Figure S8**: $^{1}$H-$^{13}$C HSQC spectra of Poly(DADMP)TFSI (left) and poly(DADMP)FSI (right).*

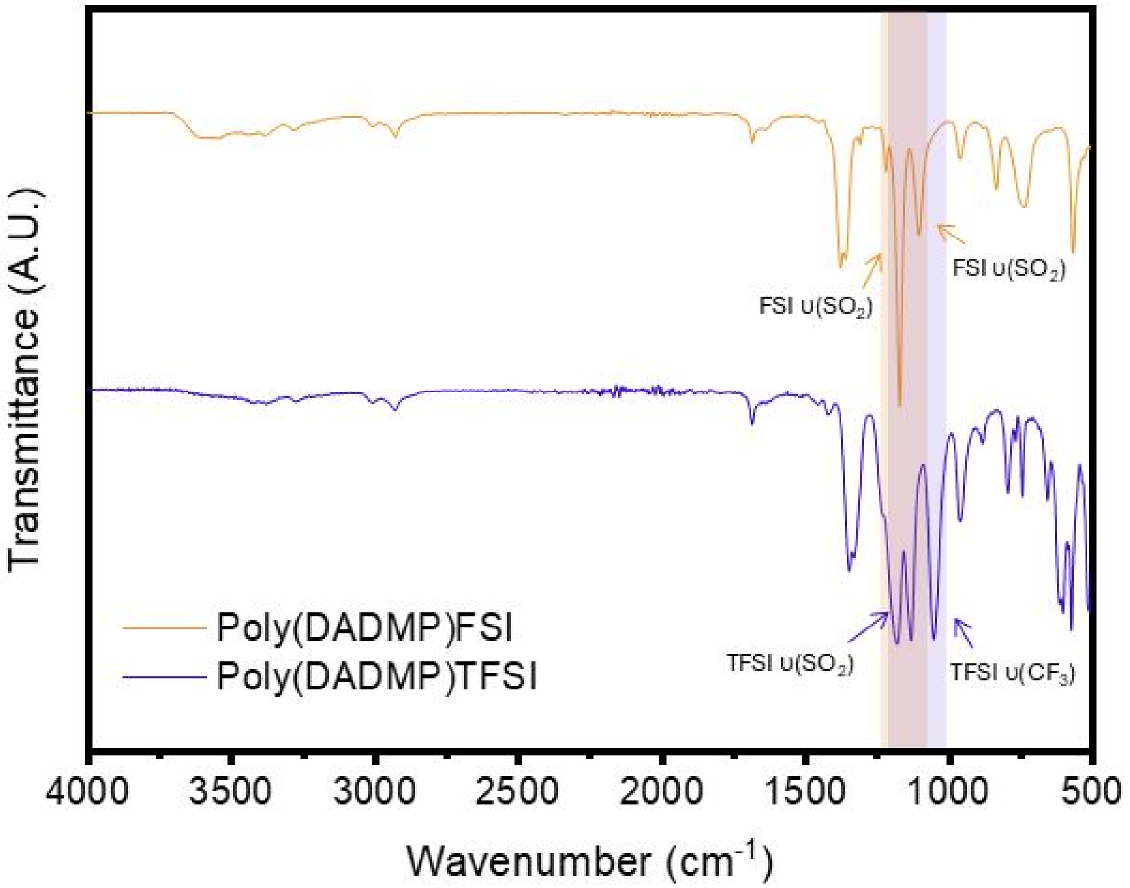


***Figure S9**: Full FTIR spectras of Poly(DADMP)FSI and Poly(DADMP)TFSI*

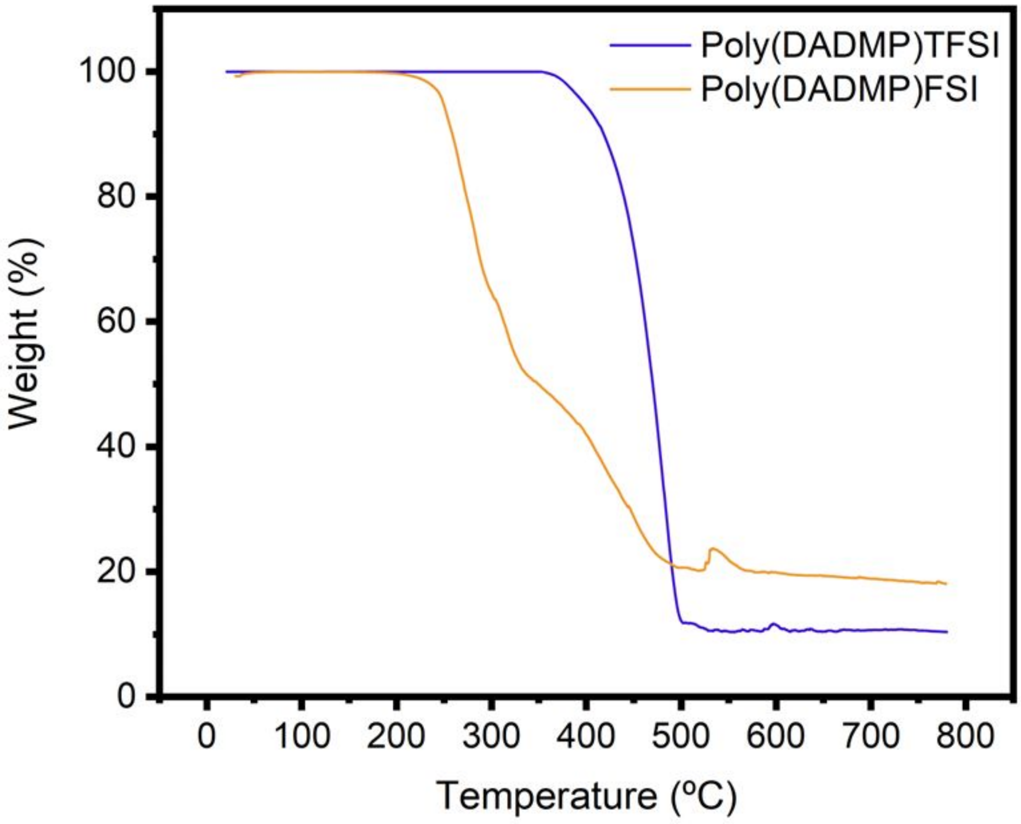


***Figure S10**: TGA of Poly(DADMP)FSi and Poly(DADMP)TFSI at scan rate of 10 °C/min.*

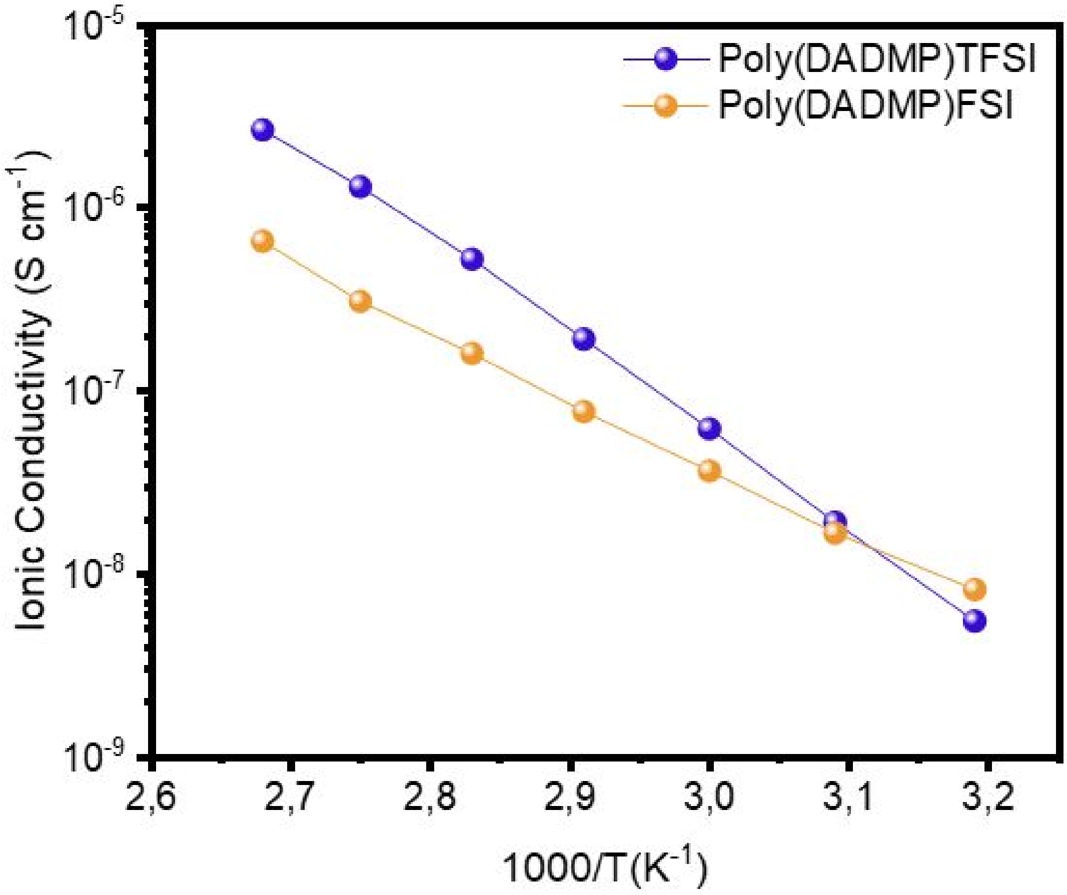


***Figure S11**: Ionic conductivity of the Poly(DADMP)FSI and Poly(DADMP)TFSI homopolymers.*

**Simulation details**

**Preparation of Molecular Dynamic (MD) and Force Field (FF) generation .** A single polymer chain with a degree of polymerization of 20 was employed in this simulation. The initial polymer structure was generated using Avogadro 1.0 and optimized with the Merck Molecular Force Field 94 (MMFF94).[1] The anion geometry was optimized using Gaussian 09 at the B3LYP/6-31+G(d) level of theory. The simulation box contained 12 polymer chains and 240 anions, along with varying ratios of LiFSI salt to construct polymer-in-salt systems. The simulation box and corresponding force field files were prepared using fftool [2] and Packmol [3]. The force-field parameters for the polycations were derived from all-atom OPLS-AA oligomer parameters generated using the LigParGen online server.[4] The monomer structures differ in their torsion angles, corresponding to distinct structural minima). Detailed parameter information is provided in the Supplementary Table 1. The parameters for lithium cations, $FSI^-$, and $TFSI^-$ anions were adopted from the CL&P force field developed by Canongia Lopes and Pádua[2], which employs the same OPLS-AA functional form. Partial charges were uniformly scaled by a factor of 0.7 for all species to account for electronic polarisation effects.

**Molecular Dynamic Procedures.** All-atom molecular dynamics (MD) simulations were performed using the GROMACS 2022 software package.[6] Periodic boundary conditions were applied in all three spatial dimensions. Bond constraints involving hydrogen atoms were maintained using the LINCS algorithm.[7] Long-range electrostatic interactions were computed with the Particle-Mesh Ewald (PME) method[6], employing a real-space cutoff of 1.2 nm, a PME order of 4, and a Fourier grid spacing of 0.16 nm. van der Waals interactions were truncated at 1.2 nm, with long-range dispersion corrections applied to both energy and pressure.

Initially, all molecular structures were optimised by energy minimisation using the steepest descent algorithm, with convergence defined by a maximum force threshold of 1000 kJ $mol^{-1}$ $nm^{-1}$, following standard GROMACS procedures. A pre-equilibration process was then conducted, consisting of a 20 ns isothermal–isobaric (NPT) simulation at 600 K and 100 bar, followed by a 6 ns canonical (NVT) simulation at 1000 K and 1 bar using the V-rescale thermostat (τ□ = 0.1 ps) and the Berendsen barostat (τ□ = 0.1 ps).[8] Next, a "21-step equilibration protocol" [9] (Table S2) is conducted, which alternates between NVT and NPT ensembles under high-temperature and high-pressure conditions to overcome large energy barriers inherent to polymer systems. These additional compression steps remove unrealistic free volumes and improve the reproducibility of simulated properties.[10]

After this pre-equilibration process, each system was further equilibrated via annealing procedures using the V-rescale thermostat (τ□ = 0.5 ps) and the Berendsen barostat (τ□ = 2.0 ps). The temperature was initially held at 353 K, followed by stepwise heating to 450 K, 500 K, 600 K, and 700 K, before being gradually cooled back to 353 K Subsequently, an additional 200 ns NPT equilibration was performed at 353 K using the leap-frog integrator (integrator = md) with a 1 fs time step, employing the V-rescale thermostat (τ□ = 0.1 ps) and the Berendsen barostat (τ□ = 1.0 ps). Then, a 100 ns production NPT simulation was carried out at 353 K using the leap-frog integrator with a 1 fs time step. Temperature was controlled using a Nosé–Hoover thermostat (τ□ = 1 ps), and pressure was maintained at 1 bar using a Parrinello–Rahman barostat (τ□ = 5.0 ps, compressibility = $4.5 \times 10^{-5}$ $bar^{-1}$).Finally, a 100 ns NVT simulation at 353 K was conducted to analyse ion diffusion behaviour, with the temperature controlled by the Nosé–Hoover thermostat (τ□ = 1 ps).

***Supplementary Table 2**: The detail of force field for MD simulations*

| **Atom** | **charge** | $\sigma$(Å) | **Eps( kJ mol$^{-1}$)** | **Atom** | **charge** | $\sigma$(Å) | **Eps ( kJ mol$^{-1}$)** |
|---|---|---|---|---|---|---|---|
| **PDADMP** | | | | | | | |
| P4 | 1.316 | 3.74 | 0.8368 | **FSI** | | | |
| C1 | -0.476 | 3.5 | 0.27614 | S | 0.714 | 3.55 | 1.046 |
| C2 | -0.42 | 3.5 | 0.27614 | N | -0.462 | 3.25 | 0.71128 |
| C3 | -0.07 | 3.5 | 0.27614 | O | -0.371 | 3.15 | 0.83736 |
| C4 | -0.098 | 3.5 | 0.27614 | F | -0.091 | 3.118 | 0.2554 |
| C5 | -0.147 | 3.5 | 0.27614 | **TFSI** | | | |
| H1 | 0.112 | 2.5 | 0.12552 | C | 0.245 | 3.5 | 0.27614 |
| H2 | 0.105 | 2.5 | 0.12552 | S | 0.714 | 3.55 | 1.046 |
| H3 | 0.07 | 2.5 | 0.12552 | N | -0.462 | 3.25 | 0.71128 |
| H4 | 0.07 | 2.5 | 0.12552 | O | -0.371 | 3.15 | 0.83736 |
| H5 | 0.063 | 2.5 | 0.12552 | F | -0.112 | 3.118 | 0.2554 |
| BONDS | Length (Å) | K | | **Li ion** | | | |
| **PDADMP** | | | | Li | 0.7 | 2.126 | 0.07648 |
| P-C | 1.82 | 1774 | | **ANGLES** | Deg ($\theta$) | K | |
| H-C | 1.09 | 2845.1 | | **PDADMP** | | | |
| C-C | 1.529 | 2242.6 | | H-C-P | 109.5 | 343.1 | |
| **FSI** | | | | C-P-C | 109.5 | 376.6 | |
| S-O | 1.437 | 5331 | | P-C-C | 109.5 | 359.8 | |
| N-S | 1.57 | 3137 | | C-C-C | 112.7 | 488.5 | |

| | | | |
|---|---|---|---|
| F-S | 1.575 | 1879 | |
| **TFSI** | | | |
| F-C | 1.323 | 3698 | |
| C-S | 1.818 | 1950 | |
| S-O | 1.437 | 5331 | |
| N-S | 1.57 | 3137 | |
| DIHEDRALS | v1 | v2 | v3 |
| **PDADMP** | | | |
| C-C-C-C | 5.4392 | -0.2092 | 0.8368 |
| C-C-C-H | 0 | 0 | 1.2552 |
| H-C-C-H | 0 | 0 | 1.2552 |
| C-P-C-H | 0 | 0 | 1.2552 |
| C-P-C-C | 4.184 | -2.092 | 2.092 |
| H-C-C-P | 0 | 0 | 1.2552 |
| C-C-C-P | 7.163 | -2.092 | 2.7782 |
| **FSI** | | | |
| O-S-N-S | 0 | 0 | -0.015 |
| F-S-N-S | 11.445 | -15.186 | -3.212 |
| **TFSI** | | | |
| O-S-C-F | 0 | 0 | 1.451 |
| N-S-C-F | 0 | 0 | 1.322 |
| O-S-N-S | 0 | 0 | -0.015 |
| S-N-S-C | 32.773 | -10.42 | -3.195 |

| | | |
|---|---|---|
| C-C-H | 110.7 | 313.8 |
| H-C-H | 107.8 | 276.1 |
| **FSI** | | |
| O-S-O | 118.5 | 969 |
| N-S-O | 113.6 | 789 |
| S-N-S | 125.6 | 671 |
| F-S-O | 104.1 | 1077 |
| F-S-N | 103 | 902 |
| **TFSI** | | |
| F-C-F | 107.1 | 781 |
| F-C-S | 111.7 | 694 |
| O-S-O | 118.5 | 969 |
| C-S-O | 102.6 | 870 |
| N-S-O | 113.6 | 789 |
| N-S-C | 103.5 | 764 |
| S-N-S | 125.6 | 671 |

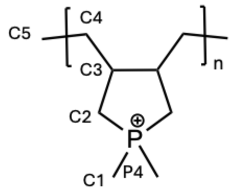


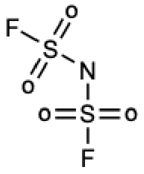

FSI

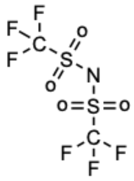

TFSI

***Supplementary Table 3***: *The detail of All-atomic Molecular Dynamic (MD) simulations*

| Step | Ensemble | Temperature (K) | Pressure (bar) | Timestep (fs) | Thermostat | Barostat | Time (ps) |
|---|---|---|---|---|---|---|---|
| 1 | Minimization (steepest descent) | N/A | N/A | N/A | N/A | N/A | N/A |
| 2 | NPT | 600 | 100 | 1 | V-rescale | Berendsen | 20000 |
| 3 | NVT | 1000 | 1 | 1 | V-rescale | | 600 |
| 4 | NVT | 1000 | 1 | 1 | V-rescale | | 50 |
| 5 | NVT | 353 | 1 | 1 | V-rescale | | 50 |
| 6 | NPT | 353 | 1000 | 1 | V-rescale | Berendsen | 50 |
| 7 | NVT | 1000 | 1 | 1 | V-rescale | | 50 |
| 8 | NVT | 353 | 1 | 1 | V-rescale | | 100 |
| 9 | NPT | 353 | 30000 | 1 | V-rescale | Berendsen | 50 |
| 10 | NVT | 1000 | 1 | 1 | V-rescale | | 50 |
| 11 | NVT | 353 | 1 | 1 | V-rescale | | 100 |
| 12 | NPT | 353 | 50000 | 1 | V-rescale | Berendsen | 50 |
| 13 | NVT | 1000 | 1 | 1 | V-rescale | | 50 |
| 14 | NVT | 353 | 1 | 1 | V-rescale | | 100 |
| 15 | NPT | 353 | 25000 | 1 | V-rescale | Berendsen | 5 |
| 16 | NVT | 1000 | 1 | 1 | V-rescale | | 5 |
| 17 | NVT | 353 | 1 | 1 | V-rescale | | 10 |
| 18 | NPT | 353 | 5000 | 1 | V-rescale | Berendsen | 5 |
| 19 | NVT | 1000 | 1 | 1 | V-rescale | | 5 |
| 20 | NVT | 1000 | 1 | 1 | V-rescale | | 10 |
| 21 | NPT | 353 | 500 | 1 | V-rescale | Berendsen | 5 |
| 22 | NVT | 1000 | 1 | 1 | V-rescale | | 5 |

| | | | | | | | |
|---|---|---|---|---|---|---|---|
| 23 | NVT | 353 | 1 | 1 | V-rescale | | 10 |
| 24 | NPT | 353 | 1 | 1 | V-rescale | Berendsen | 800 |
| 25 | NPT Annealing | 353, 450, 500, 600, 700, 600, 500, 470 450, 353 | 1 | 1 | V-rescale | Berendsen | 600, 200, 100, 100, 7600, 400,,600,200,500 |
| 26 | NPT | 353 | 1 | 1 | Nose-Hoover | Parrinello-Rahman | 50000 |
| 27 | NVT | 353 | 1 | 1 | Nose-Hoover | | 100000 |

**Protocol of polymer glass transition temperature ($T_g$) simulation:** The glass-transition temperature (Tg) of polymers can be determined from the thermal expansivity (α = dV/dT). Therefore, the volume–temperature (VT) profiles were calculated through a series of NPT simulations.[10,11] Each equilibrated system was heated to 600 K and subsequently cooled to 100 K in a stepwise manner with a 25 K interval at a average cooling rate of 1 K $ns^{-1}$.[12] The Tg value was extracted from the intersection of two linear fits of V-T profile in both the glassy and rubbery regimes. The quality of the linear regression was evaluated using the coefficient of determination ($R^2$), which was required to exceed 0.99, with $R^2 = 1$ indicating a perfect fit.

**Mean-Squared Displacement (MSD):** The MSD was calculated at 353 K for 60 ns using a 100 ns NVT MD trajectory to ensure the linearity. The self-diffusion coefficient (D) was subsequently obtained from the last linear region of the MSD profile according to Equation (1):

$$D = \frac{1}{N}\sum_{i=1}^{N} \frac{\Delta MSD(t)}{6\Delta t} \quad \text{Eq. 1}$$

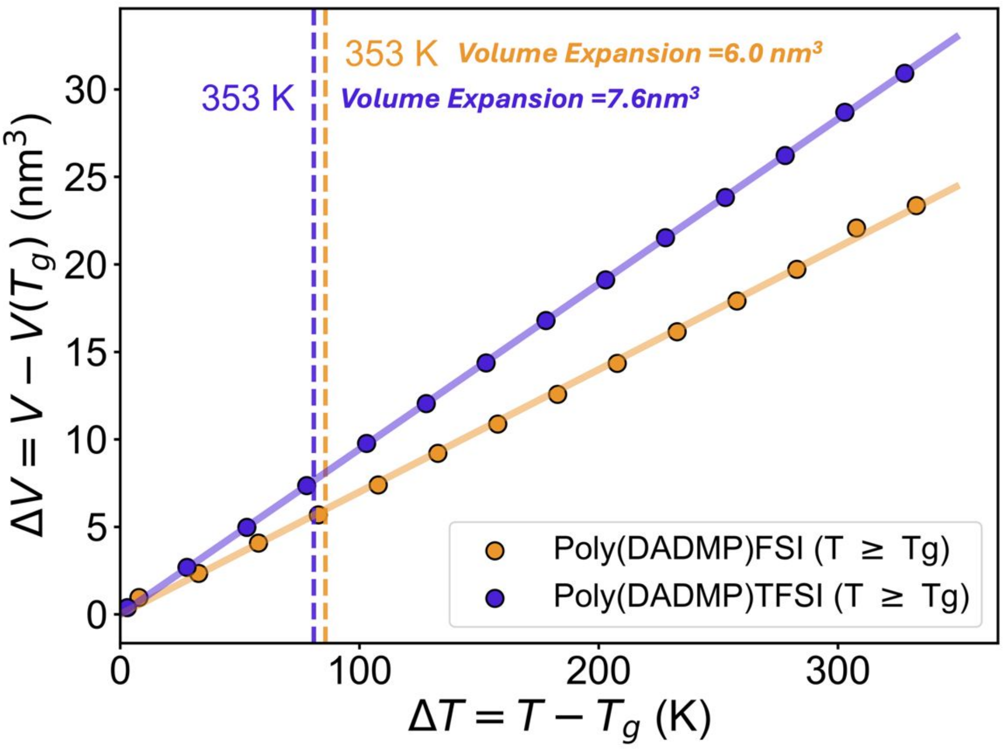


***Figure S12:*** *Volume expansion of PDADMP FSI and PDADMP TFSI systems above the polymer* $T_g$

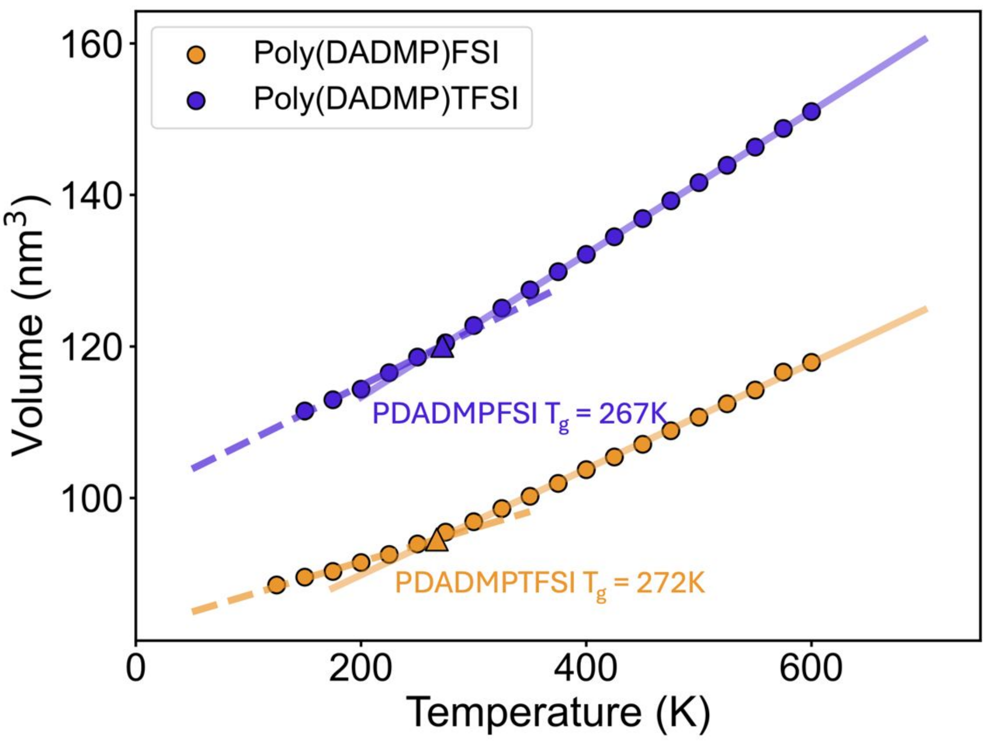


***Figure S13:*** *Simulated $T_g$ PDADMP FSI and PDADMP TFSI systems*

***Solid-state polymer electrolytes (SSPE)s preparation and characterization***

The polymers and salt were dissolved in dry acetonitrile inside a glovebox and stirred overnight. The solvent was then removed under vacuum conditions (rotavapor), and the material was thoroughly dried under a high vacuum for two days with temperature ($8 \times 10^{-8}$ mbar and 60 °C), resulting in a solid-viscous orange substance. In this paper, the nomenclature for naming the electrolytes is based on the polymer used (Poly(DADMP)TFSI and Poly(DADMP)FSI); the salt used will always be LiFSI, and the ratio of polymer to salt is specified. For example, Poly(DADMP)TFSI:LiFSI 1:2 refers to a material composed of 1 mol of polymer Poly(DADMP)TFSI and 2 mols of LiFSI.

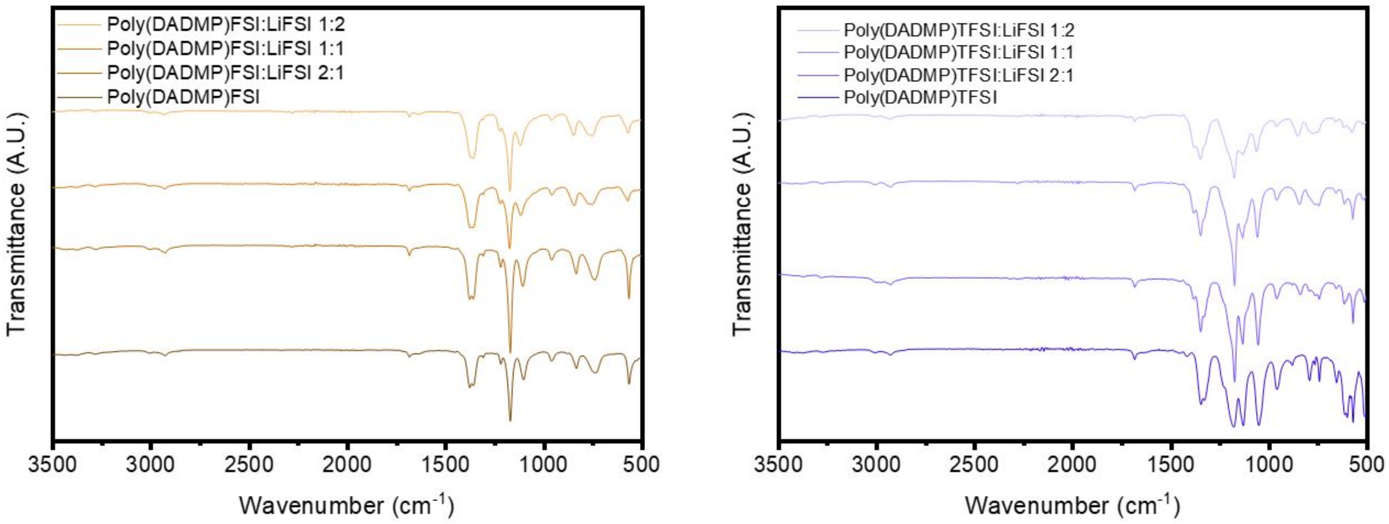


***Figure S14****: Full FTIR spectra on the Poly(DADMP)FSI:LiFSI and Poly(DADMP)TFSI:LiFSI at different ratios polymer:salt.*

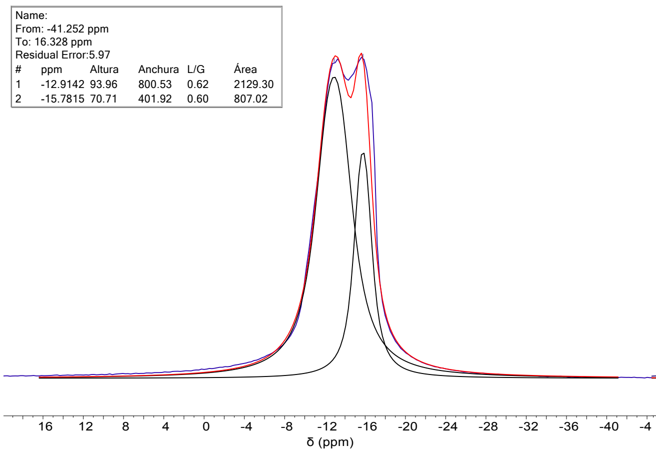
Name:
From: -41.252 ppm
To: 16.328 ppm
Residual Error:5.97
# ppm Altura Anchura L/G Área
1 -12.9142 93.96 800.53 0.62 2129.30
2 -15.7815 70.71 401.92 0.60 807.02
δ (ppm)

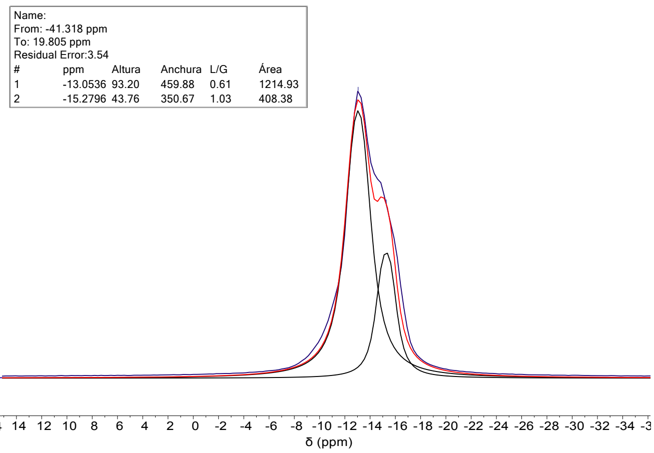
Name:
From: -41.318 ppm
To: 19.805 ppm
Residual Error:3.54
# ppm Altura Anchura L/G Área
1 -13.0536 93.20 459.88 0.61 1214.93
2 -15.2796 43.76 350.67 1.03 408.38
δ (ppm)

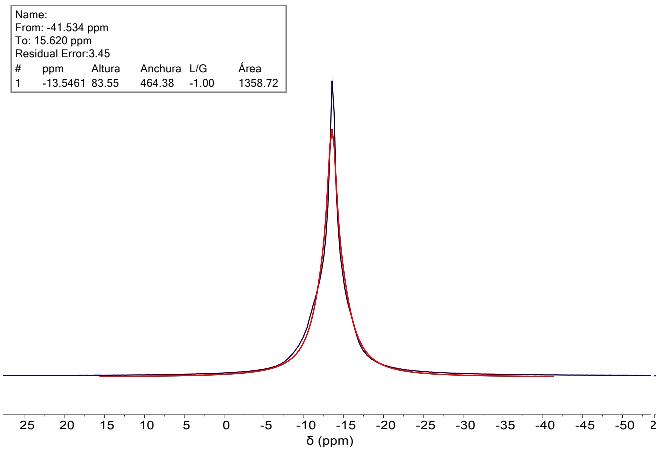
Name:
From: -41.534 ppm
To: 15.620 ppm
Residual Error:3.45
# ppm Altura Anchura L/G Área
1 -13.5461 83.55 464.38 -1.00 1358.72
δ (ppm)

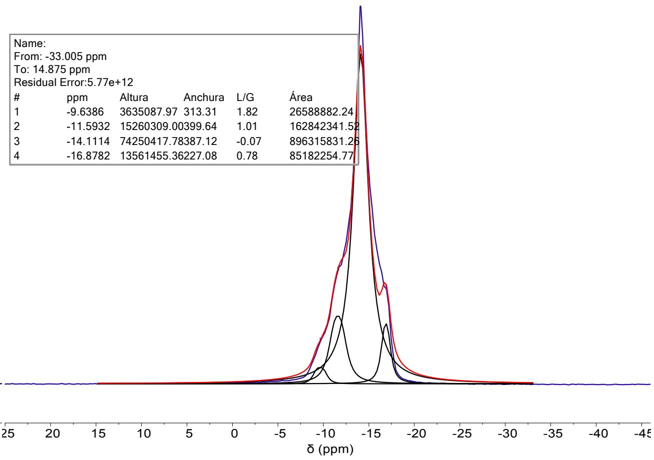
Name:
From: -33.005 ppm
To: 14.875 ppm
Residual Error:5.77e+12
# ppm Altura Anchura L/G Área
1 -9.6386 3635087.97 313.31 1.82 26588882.24
2 -11.5932 15260309.00399.64 1.01 162842341.52
3 -14.1114 74250417.78387.12 -0.07 896315831.26
4 -16.8782 13561455.36227.08 0.78 85182254.77
δ (ppm)

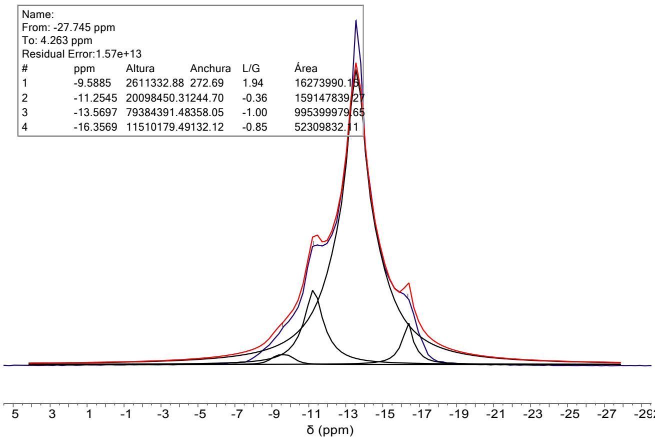
Name:
From: -27.745 ppm
To: 4.263 ppm
Residual Error:1.57e+13
# ppm Altura Anchura L/G Área
1 -9.5885 2611332.88 272.69 1.94 16273990.18
2 -11.2545 20098450.31244.70 -0.36 159147839.27
3 -13.5697 79384391.48358.05 -1.00 995399979.65
4 -16.3569 11510179.49132.12 -0.85 52309832.11
δ (ppm)

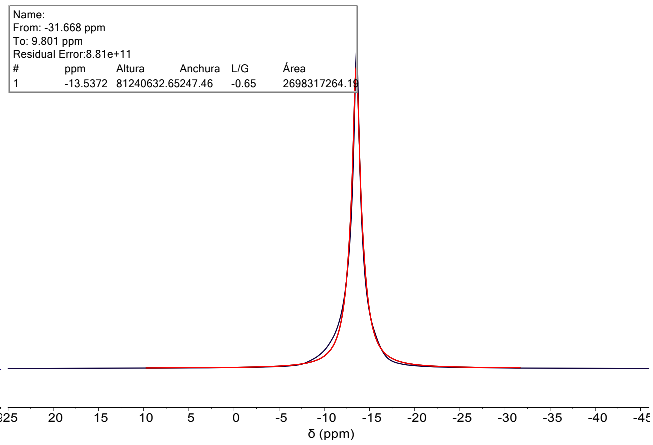
Name:
From: -31.668 ppm
To: 9.801 ppm
Residual Error:8.81e+11
# ppm Altura Anchura L/G Área
1 -13.5372 81240632.65247.46 -0.65 2698317264.19
δ (ppm)

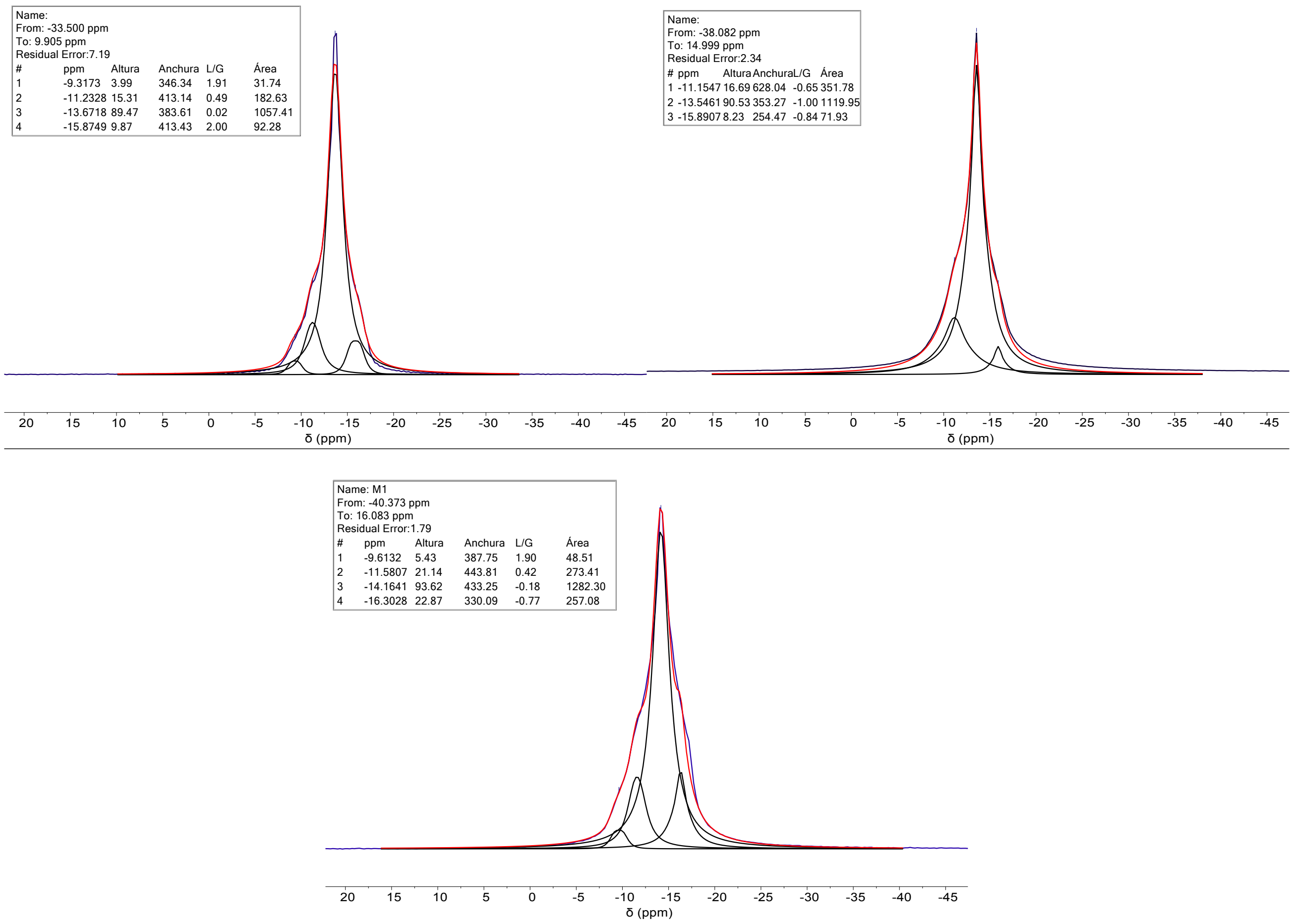


***Figure S15:*** *Deconvolution calculations of the $^{7}Li$ solid-state NMR*

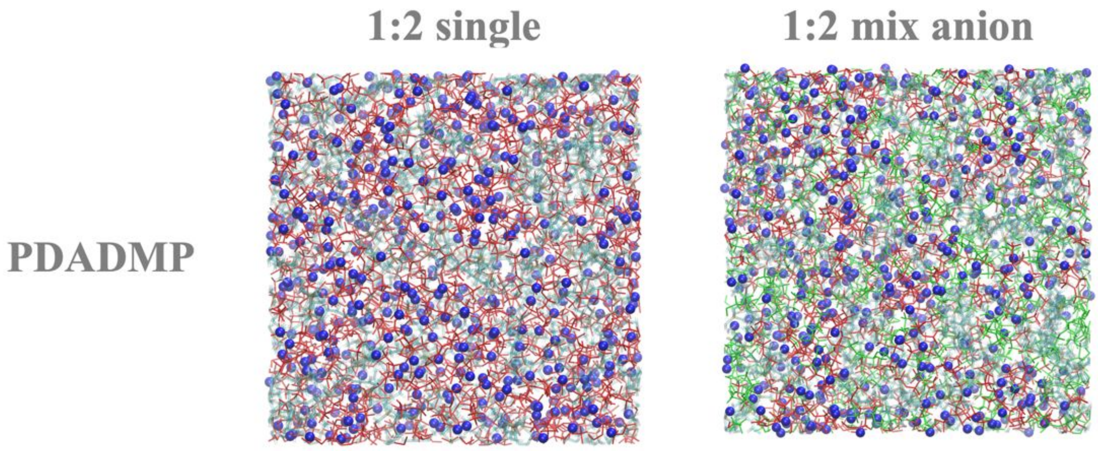


***Figure S16:*** *Simulation boxes of PDADMP FSI : LiFSI=1:2 system and PDADMP TFSI : LiFSI=1:2 system*

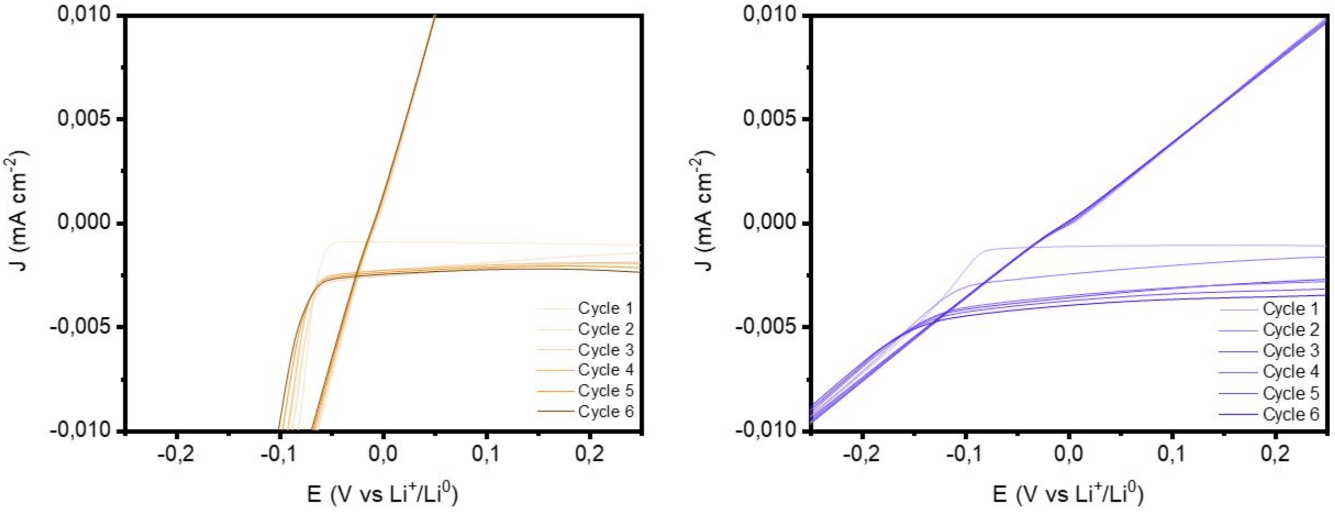


***Figure S17**: Cyclic voltammetry zoom of the starting plating zone.*

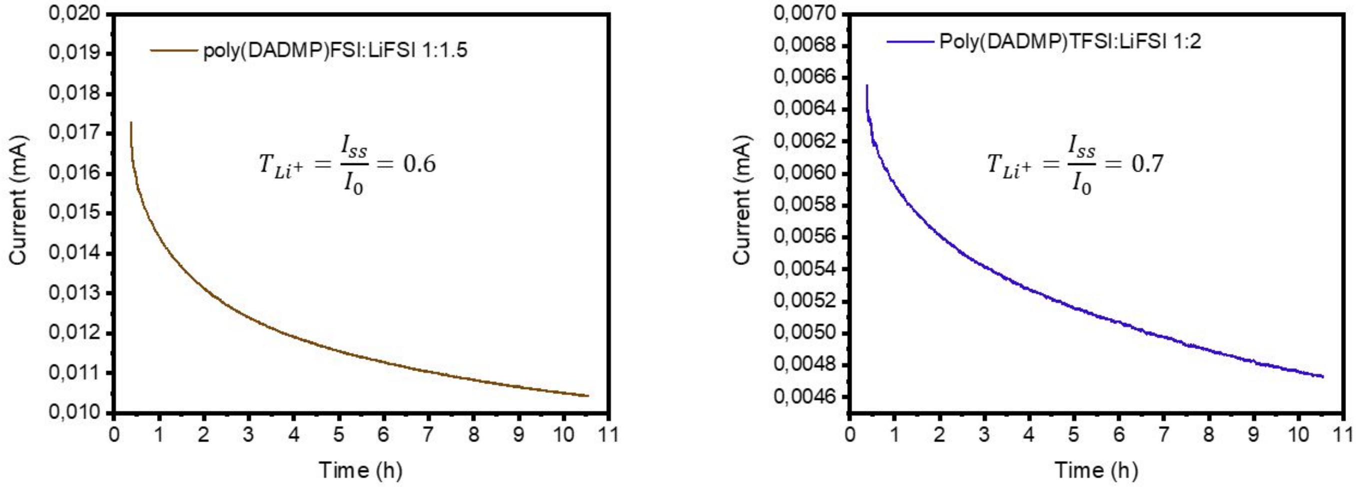


***Figure S18**: Chronoamperometry test performed at 60 °C on Li/Li cell with Poly(DADMP)FSI:LIFSI 1:1.5 (left) and Poly(DADMP)TFSI:LIFSI 1:2 (right) to evaluate the steady state and initial current values and extrapolate the $Li^+$ transference number ($t_{Li+}$).*

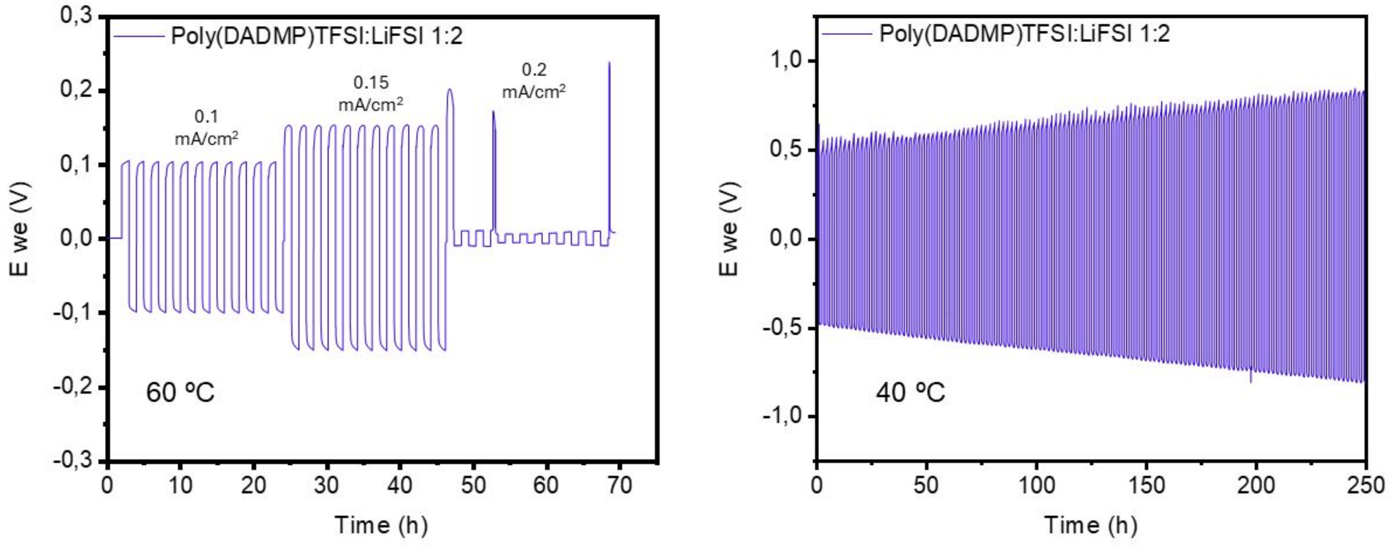


*Figure S19: C-rate experiment of Poly(DADMPT)FSI:LIFSI 1:2 electrolyte (left); Li|Li cycling test at 40 °C of Poly(DADMP)TFSI:LIFSI 1:2 elelctrolyte (right)*

***Supplementary Table 4****: Solubility table summarize of Poly(DADMP)FSI and Poly(DADMP)TFSI*

| Sample | Molecular weight (g/mol) | Tg onset (ºC) | Ionic conductivity at 30ºC (S $cm^{-1}$) | Ionic conductivity at 70 ºC (S $cm^{-1}$) | Overvoltage in Li\|Li cycling at 60 ºC (after 100 cycles) (V) | Overvoltage in Li\|Li cycling at 40 ºC (after 100 cycles) (V) |
|---|---|---|---|---|---|---|
| **Poly(DADMP)FSI:LiFSI 1:1,5** | 40879 | -58 | 1.5 $x10^{-4}$ | 9.9 $x10^{-4}$ | 0.07 | 0.18 |
| **Poly(DADMP)TFSI:LiFSI 1:2** | 40879 | -24 | 1.1 $x10^{-5}$ | 1.7 $x10^{-4}$ | 0.15 | 0.66 |

## REFERENCES


1 Hanwell, M. D. *et al.* Avogadro: an advanced semantic chemical editor, visualization, and analysis platform. *J. Cheminform.* **4**, 17, doi:10.1186/1758-2946-4-17 (2012).

2 Canongia Lopes, J. N. & Pádua, A. A. H. CL&P: A generic and systematic force field for ionic liquids modeling. *Theor. Chem. Acc.* **131**, 1129, doi:10.1007/s00214-012-1129-7 (2012).

3 Martínez, L., Andrade, R., Birgin, E. G. & Martínez, J. M. PACKMOL: A package for building initial configurations for molecular dynamics simulations. *J. Comput. Chem.* **30**, 2157-2164, doi:https://doi.org/10.1002/jcc.21224 (2009).

4 Doherty, B., Zhong, X., Gathiaka, S., Li, B. & Acevedo, O. Revisiting OPLS Force Field Parameters for Ionic Liquid Simulations. *J. Chem. Theory Comput.* **13**, 6131-6145, doi:10.1021/acs.jctc.7b00520 (2017).

5 Wang, X. *et al.* Poly (ionic liquid) s-in-salt electrolytes with co-coordination-assisted lithium-ion transport for safe batteries. *Joule* **3**, 2687-2702 (2019).

6 Van Der Spoel, D. *et al.* GROMACS: fast, flexible, and free. *J. Comput. Chem.* **26**, 1701-1718 (2005).

7 Krieger, E. & Vriend, G. New ways to boost molecular dynamics simulations. *J. Comput. Chem.* **36**, 996-1007, doi:https://doi.org/10.1002/jcc.23899 (2015).

8 Marioni, N. *et al.* Ion and Water Dynamics in the Transition from Dry to Wet Conditions in Salt-Doped PEG. *ACS Macro Lett.* **13**, 341-347, doi:10.1021/acsmacrolett.4c00046 (2024).

9 Hofmann, D., Fritz, L., Ulbrich, J., Schepers, C. & Böhning, M. Detailed-atomistic molecular modeling of small molecule diffusion and solution processes in polymeric membrane materials. *Macromol. Theory Simul.* **9**, 293-327, doi:https://doi.org/10.1002/1521-3919(20000701)9:6<293::AID-MATS293>3.0.CO;2-1 (2000).

10 Abbott, L. J., Hart, K. E. & Colina, C. M. Polymatic: a generalized simulated polymerization algorithm for amorphous polymers. *Theor. Chem. Acc.* **132**, 1334, doi:10.1007/s00214-013-1334-z (2013).

11 Kondou, S. *et al.* Poly(Ionic Liquid) Electrolytes at an Extreme Salt Concentration for Solid-State Batteries. *Journal of the American Chemical Society* **146**, 33169-33178, doi:10.1021/jacs.4c12616 (2024).

12 Gudla, H. & Zhang, C. How to Determine Glass Transition Temperature of Polymer Electrolytes from Molecular Dynamics Simulations. *The Journal of Physical Chemistry B* **128**, 10537-10540, doi:10.1021/acs.jpcb.4c06018 (2024).
